# A low dose of RBD and TLR7/8 agonist displayed on influenza virosome particles protects rhesus macaque against SARS-CoV-2 challenge

**DOI:** 10.1038/s41598-023-31818-y

**Published:** 2023-03-28

**Authors:** Gerrit Koopman, Mario Amacker, Toon Stegmann, Ernst J. Verschoor, Babs E. Verstrepen, Farien Bhoelan, Denzel Bemelman, Kinga P. Böszörményi, Zahra Fagrouch, Gwendoline Kiemenyi-Kayere, Daniella Mortier, Dagmar E. Verel, Henk Niphuis, Roja Fidel Acar, Ivanela Kondova, Yolanda S. Kap, Willy M. J. M. Bogers, Petra Mooij, Sylvain Fleury

**Affiliations:** 1 Department of Virology, Biomedical Primate Research Centre (BPRC), Rijswijk, The Netherlands; 2 Mymetics SA, 4 Route de La Corniche, 1066 Epalinges, Switzerland; 3 Department for BioMedical Research DBMR, Department of Pulmonary Medicine, Inselspital, Bern University Hospital, University of Bern, 3008 Bern, Switzerland; 4 Mymetics BV, JH Oortweg 21, 2333 CH Leiden, The Netherlands; 5 Animal Science Department, Biomedical Primate Research Centre (BPRC), Rijswijk, The Netherlands

**Keywords:** Immunology, Infectious diseases

## Abstract

Influenza virosomes serve as antigen delivery vehicles and pre-existing immunity toward influenza improves the immune responses toward antigens. Here, vaccine efficacy was evaluated in non-human primates with a COVID-19 virosome-based vaccine containing a low dose of RBD protein (15 µg) and the adjuvant 3M-052 (1 µg), displayed together on virosomes. Vaccinated animals (n = 6) received two intramuscular administrations at week 0 and 4 and challenged with SARS-CoV-2 at week 8, together with unvaccinated control animals (n = 4). The vaccine was safe and well tolerated and serum RBD IgG antibodies were induced in all animals and in the nasal washes and bronchoalveolar lavages in the three youngest animals. All control animals became strongly sgRNA positive in BAL, while all vaccinated animals were protected, although the oldest vaccinated animal (V1) was transiently weakly positive. The three youngest animals had also no detectable sgRNA in nasal wash and throat. Cross-strain serum neutralizing antibodies toward Wuhan-like, Alpha, Beta, and Delta viruses were observed in animals with the highest serum titers. Pro-inflammatory cytokines IL-8, CXCL-10 and IL-6 were increased in BALs of infected control animals but not in vaccinated animals. Virosomes-RBD/3M-052 prevented severe SARS-CoV-2, as shown by a lower total lung inflammatory pathology score than control animals.

## Introduction

The severe acute respiratory syndrome coronavirus 2 (SARS-CoV-2)^1–3^ is an airborne pathogen^4,5^ responsible for the pandemic coronavirus disease of 2019 (COVID-19)^6,7^. It enters the body primarily through the upper respiratory tract (nasal cavities, mouth, and throat) but the primary SARS-CoV-2 replication site is the nasal tissue^8^ from where it spreads to the lungs and other organs and can also infect the central nervous system^9^.

SARS-CoV‐2 uses its receptor binding domain (RBD) present in the spike (S) viral membrane glycoprotein^10^ for binding to the cellular receptor angiotensin-converting enzyme 2 (ACE2)^11,12^ for infecting cells^13^. The broad tissue distribution of the ACE2^14^ may explain the numerous organs susceptible to SARS-CoV-2 infection. Furthermore, isolation and characterization of neutralizing monoclonal antibodies from SARS-CoV-2 convalescent patients confirm that RBD and S are key targets for blocking infection^15–20^, indicating that RBD and S are promising vaccinal antigens. It was postulated that SARS-CoV-2 vaccines delivered intramuscularly for eliciting protective antibodies should prevent massive virus replication and tissue damage in the lungs. This protection is thought to be due in part by the ability of vaccine-induced circulating antibodies to reach the highly vascularized lung tissues and the transport of IgG across the mucosal lung tissue into the lumen by the neonatal Fc receptor (FcRn)^21,22^.

Using different vaccinal antigens and vaccine technologies^23–34^ numerous different vaccine products against COVID-19 have been evaluated between 2020 and 2022. The main current vaccines are: mRNA vaccines (BNT16b2/Comirnaty, mRNA-1273/Spikevax, CVnCoV), adenoviral vector vaccines (ChAdOx1/Vaxzevria or Covishield, Ad26.COV2.S, Ad5-nCoV/Convidecia, Ad5-Ad26/Sputnik V, Ad26/Sputnik V Light), peptide based vaccine (EpiVacCorona), inactivated vaccines (CoronaVac, BBIBP-CorV, WIBP-CorV BBV152/Covaxin, VLA2001, COVIran Barekat), S-based protein vaccines (NVX-CoV2373, VLP-S plant derived/Covifenz, CoV2 preS dTM**/**VidPrevtyn Beta, SCB-2019), RBD-based protein vaccines (Corbevax, ZF2001/Zifivax, CIGB-66/Abdala, UB-612 with S1-RBD with T cell epitopes from S2, membrane and nuclear protein), and RBD conjugated vaccines (FINLAY-FR-1/2 or Soberana 01/02). Although the antibody titer level for achieving protection is not yet determined, these prophylactic COVID-19 vaccines were successful in preventing disease complications, hospitalizations and death. However, the vaccine-induced antibody protective immunity seems to be short-lived^35–38^, as reported by rapid drops of antibody titers within 4–6 months post-vaccination.

Furthermore, developing vaccines offering effective broad cross-strain protection is hampered by high genome mutation rates that are common to RNA viruses^39,40^ as also reported for SARS-CoV-2^41,42^. Mutations result in amino acid changes in various viral proteins, including the S or RBD antigen that can significantly reduce to different degrees the recognition by neutralizing antibodies induced during natural infection or vaccination^43–47^. This evolution/selection process allows the emergence of new virus variants that escape antibody recognition, and the Omicron B.1.1.529 variant is a good example with 37 mutations located in S protein^48^. Therefore, the future vaccines targeting conserved S or RBD regions and/or combining antigens of different virus strains may hold better cross-strain protection. Due to rapid waning immunity, combined with the rapid emergence of new variants, regular boosts may be necessary for preserving the population protection by antibodies.

According to the World Health Organization (WHO) website dedicated to the COVID-19 vaccine tracker, there are about 380 vaccines under investigation. By end of 2022, about 172 vaccines are in clinical development, and near 200 vaccine candidates are in pre-clinical development with about 80 candidates that are based on proteins and 20 using virus-like particles (VLPs) for displaying proteins, indicating that subunit vaccines are still an effective platform for the development of safe COVID-19 vaccines and can contribute to diversify the vaccine pipeline, with a demonstrated good tolerability and safety profile.

Here, we report the development of a subunit COVID-19 vaccine based on recombinant SARS-CoV-2 RBD proteins produced in yeast^49,50^ that are displayed on influenza virosomes acting as antigen carriers^51–53^, together with membrane anchored 3M-052 as TLR7/8 agonist. As known in cell membrane biology, the proteins present in a fluid lipid membrane are free to move in *cis* and/or rotate on their axis, and similarly at the virosome lipid membrane surface, which may facilitate the access to antigen epitopes. Virosomes have a mean diameter of 80–120 nm, they are devoid of nucleic acid and are non-infectious, and they belong to the enveloped virus-like particles^54,55^. Influenza virosomes are constituted of synthetics lipids and viral components derived from the influenza membrane, such as the hemagglutinin (HA) and neuraminidase (NA) glycoprotein that serve as a source of universal T-helper epitopes for the lipid-anchored vaccinal antigen^51–53^. As opposed to most VLPs that are mixed with 1–2 adjuvants for improving their immunogenicity^55,56^, which means the presence of free-form adjuvants, virosomes with lipid-anchored adjuvants into their lipid membrane have no free-form adjuvants.

More than 80 million doses of virosome-based vaccines have been safely administered, with the hepatitis A vaccine (Epaxal® commercialized in 1994)^57–60^ and the seasonal influenza vaccine (Inflexal® commercialized in 1997)^61,62^. The excellent tolerability and safety profiles of those virosomal vaccines were documented in children^59^, adults^60^ and elderlies^63^, including subjects with chronic inflammatory diseases^64,65^ or immunocompromised^61^. Clinical stage virosomal vaccines against HIV-1^66^ and *Plasmodium falciparum* malaria^67,68^ were also previously evaluated successfully in human Phase I trials. Influenza-based virosomes can be manufactured under liquid or lyophilized form for intramuscular or subcutaneous administration, as well as under liquid or thermostable spray-dried powder for intranasal delivery^66,69^. Virosomal vaccines are generally stored refrigerated, but a new thermostable spray-dried powder initially developed for our nasal HIV-1 vaccine remained stable for few months at 40 °C/75% relative humidity without compromising the vaccine bioactivity^69^.

This new manufacturing process was also applied for the development of a thermostable nasal COVID-19 vaccine, starting with an intermediate product under a liquid form subsequently downstream processed into nasal powder, which is currently under investigation. Meanwhile, we were interested to find out if the liquid version of our COVID-19 vaccine, without the excipients for spray drying but having comparable virosome-RBD and buffer composition, could induce protection, when administered intramuscularly to non-human primates (NHP). As the liquid virosomal vaccine is formulated for being compatible for nasal administration, the 3M-052 was selected as mucosal adjuvant that serves as pathogen-associated molecular patterns (PAMP) for targeting TLR7/8^70^, mimicking the RNA immunostimulatory action of many respiratory viruses (ex. Influenza, SARS-CoV-2). A very low dose of 3M-052 was strategically proposed for the design of a nasal vaccine to favor an excellent vaccine tolerance and safety profile, and to facilitate the acceptance of regular boosts in the population. This explains the low adjuvant dose present into the tested intramuscular liquid vaccine reported here. The liquid COVID-19 vaccine was found safe and well tolerated, and vaccinated macaques were protected against SARS-CoV-2 nasal/intratracheal challenge, and lung tissues had weak cell infiltrations, respective to control animals. Virosome-RBD may represent an alternative to many subunit vaccines containing much higher antigen and/or adjuvant doses, with the advantage to be also compatible for nasal immunization.

## Results

### Safety, tolerance and immunogenicity

**Figure 1:**
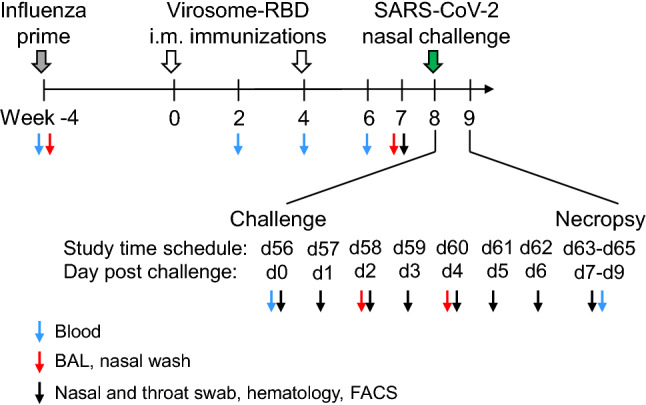
Immunization and challenge schedule in rhesus macaques. Four weeks before vaccination (week -4), Rhesus macaques of the virosome vaccine group (n = 6) and the control group (n = 4) were immunized intramuscularly (i.m.) with inactivated influenza virus particles (grey arrow) to prime immune responses against influenza specifically the hemagglutinin (HA). Subsequently, animals of the vaccine group were immunized at weeks 0 and 4 by the i.m. route with influenza-derived virosomes displaying the RBD derived from the SARS-CoV-2 Wuhan strain (open arrow). At week 8 (day 56: d56 of the study design), all animals were challenged (corresponding to d0) by the combined intranasal and intratracheal route with 1 × 10^5^ TCID_50_ of SARS-CoV-2 (green arrow). At different timepoints before and after challenge (d56 to d63-d65), diverse types of samples were collected, as indicated by color coded arrowheads. Animals were sacrificed either at day 7 (d63), 8 (d64) or 9 (d65) post-challenge.

We have evaluated a COVID-19 vaccine candidate containing 15 μg of RBD and only 1 μg of 3M-052 adjuvant per dose, which might be sufficient as a seasonal COVID-19 vaccine to be administered to a population with pre-existing SARS-CoV-2 immunity. To mimic the natural influenza immunity present in humans with anti-HA antibodies that can bind influenza virosomes, rhesus macaques were primed with inactivated influenza virus four weeks before the first vaccine administration. The serum anti-HA titers were confirmed in the serum pools collected four weeks after the first (1/53,000) and second vaccination (1/63,000) (see Fig. S1 in the supplementary materials). Subsequently, animals were vaccinated intramuscularly twice at week 0 and 4 (Fig. 1).

The vaccine was well tolerated and safe, and no local reactions were observed at the immunization site during the four days of observation following each immunization. Animals showed normal behavior and appetite, overall clinical chemistry (Table S1) and hematology parameters (Table S2) were in the normal range. We performed the systemic inflammatory cytokine profiles at 3 weeks post vaccination to better evaluate the residual mid-term effect due to vaccination. For this reason, levels of IL-1β, IL-6, IL-8, IL-10. IL-12p40, IL-17A, IL-23, GM-CSF, IFNβ, IFNγ, TNFα, CXCL10 and CCL2 were measured in serum collected two weeks after the second immunization (week 6).

**Figure 2:**
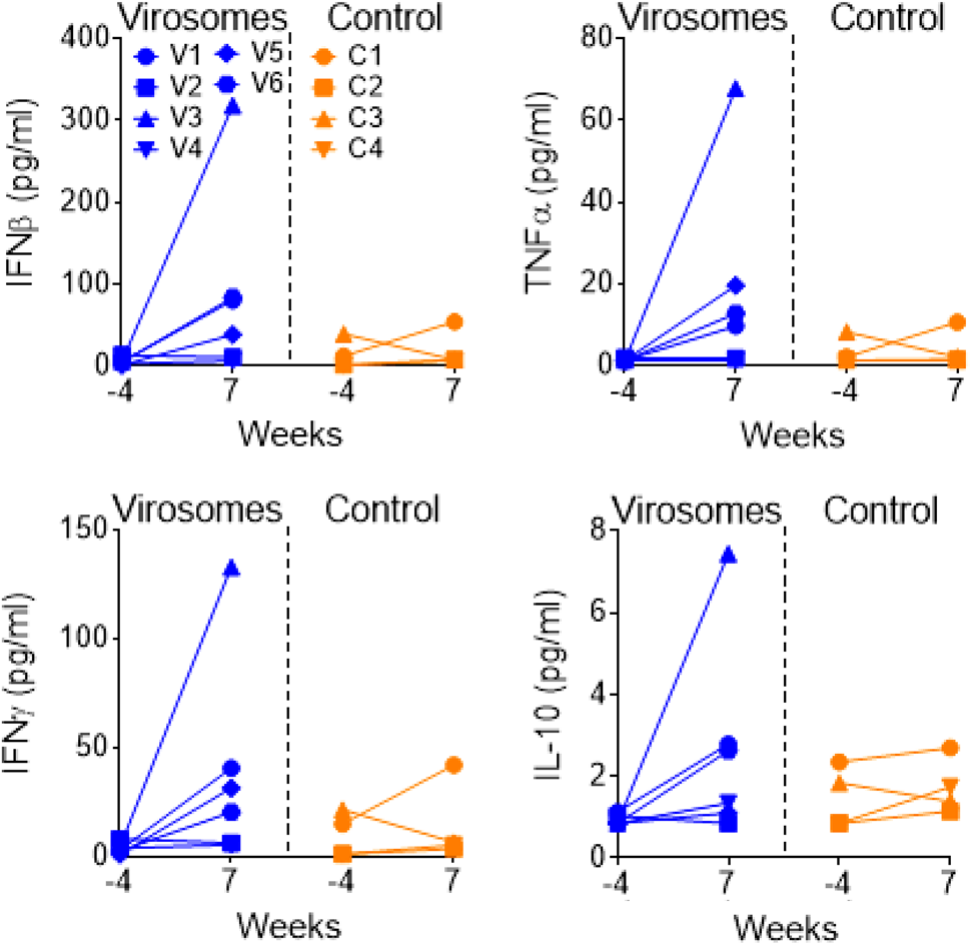
Cytokine responses in nasal washes during immunization. IFNβ (upper left panel), TNFα (upper right panel), IFNγ (lower left panel) and IL-10 (lower right panel) cytokine levels (pg/mL) measured in nasal washes, either before immunization (week -4) or three weeks after the second immunization (week 7). Macaques immunized with virosomes-RBD (V1 to V6 in blue) and control animals (C1 to C4 in orange). Each animal is represented by a different symbol. For the serum, week -4 versus week 7 (21 days after last immunization), the vaccinated group is not significantly different from the control group for IFNβ, TNFα, IFNγ and IL-10 (Mann–Whitney test). However, there is a significant increase of cytokines in nasal washes of virosome vaccinated animals (week-4 versus week 7, Mann–Whitney test) for IFNβ (*p* = 0.087), TNFα (*p* = 0.013), IFNγ (*p* = 0.087), and IL-10 (*p* = 0.433), while these increases are not observed in control animals.

None of these cytokines or chemokines remained increased in serum and were all expressed at baseline levels (not shown). However, increased levels of IFNβ, TNFα and IFNγ were observed locally in the nasal wash collected three weeks after the second immunization in four out of 6 immunized animals, and IL-10 was increased in three immunized animals (Fig. 2). Meanwhile, one of the four control animals also showed an increase in these nasal cytokine levels (Fig. 2), indicating that other factors besides immunization may trigger the same kind of responses. There was no increase in any of these cytokines or chemokines observed in BAL fluid collected three weeks after the second immunization (data not shown).

**Figure 3:**
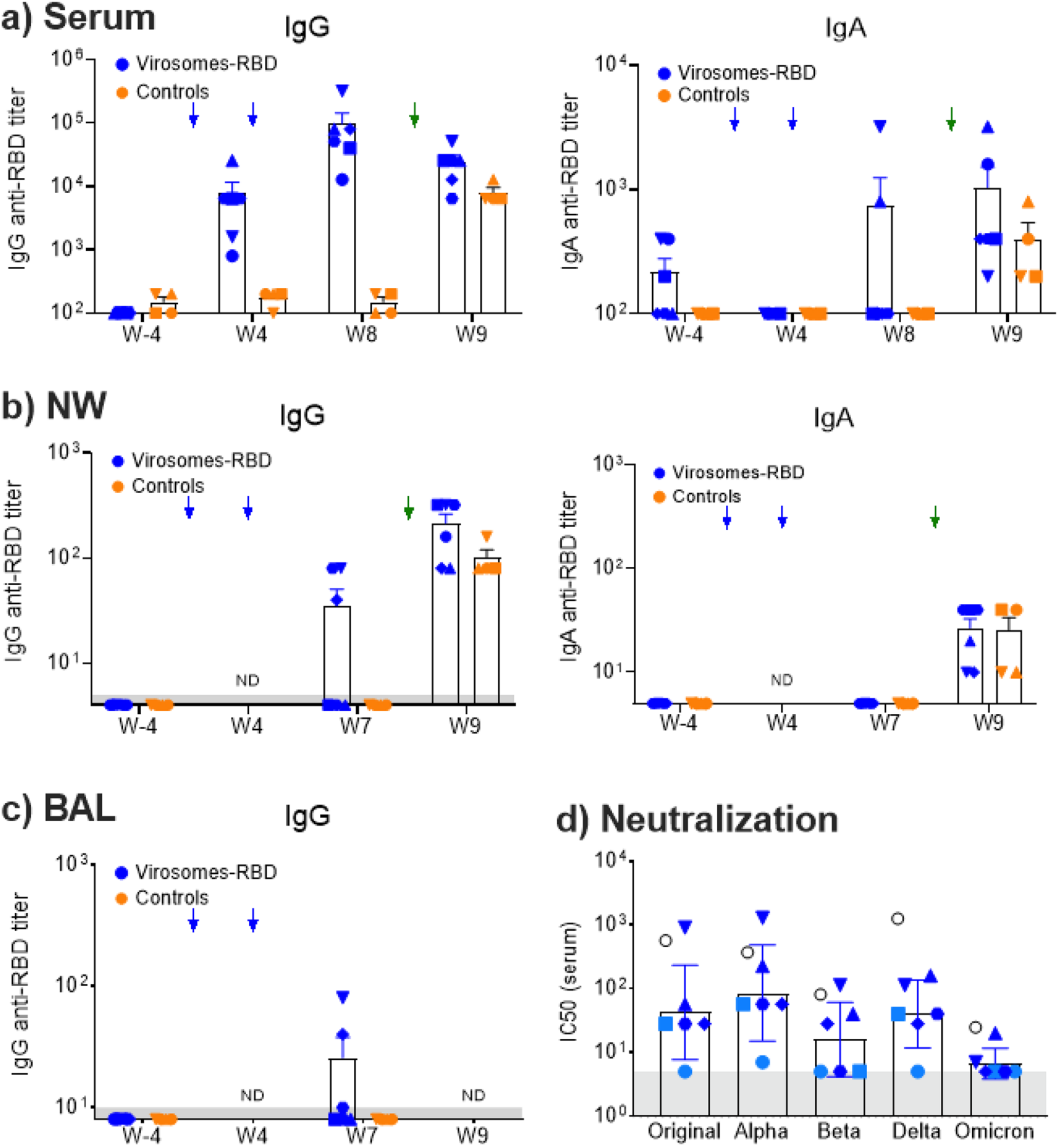
RBD specific IgG and IgA ELISA and serum neutralizing activity. (**a**) ELISA serum IgG (left graph) and IgA (right graph) antibody titers overtime toward SARS-CoV-2 RBD are shown for the vaccinated and control group. Serum IgG titers at week 4 (*p* = 0.0095) and week 8 (*p* = 0.0095) are significanlty higher in the vaccinated group than the control group, but at week 9 there is no significant difference (*p* = 0.076). (**b**) ELISA nasal wash (NW) IgG (left graph) and IgA (right graph) antibody titers overtime against SARS-CoV-2 RBD. (**c**) ELISA BAL IgG (left graph) antibody titers overtime against SARS-CoV-2 RBD. Each animal is represented by a symbol, blue for vaccinated animals and orange for control animals. Mean and SEM are shown in columns for each group of animals. Blue arrows indicate the day of immunization at week 0 and week 4, green arrow the day of the challenge at d56/week 8. For IgA in serums, NWs and BALs, there was no significant difference between the groups (*p* > 0.05). (**d**) Individual serum neutralizing antibody titers from week 8/d56 were determined by neutralization assays with five different live SARS-CoV-2 strains. The determined geomean (GM) neutralizing titers (NT) are the following: Italian strain INMI1 from 2019 as an original Wuhan-like (GM NT 42), Alpha B.1.1. 7 (GM NT 85), Beta B.1.351 (GM NT 16), Delta B.1.617.2 (GM NT 40) and Omicron B.1.1.529 (geomean NT 7). There was no significant differences for the neutralizing titers between the Wuhan-like original strain with the Beta (*p* = 0.125) and Delta strain (*p* = 0.750), but a higher neutralizing titer against the Alpha strain was observed (*p* = 0.031). However, we observe an apparent reduced neutralizing activity toward the Omicron strain, respective to the Wuhan. The closed circles and squares in light blue are animals with the lowest serum RBD titers that are also the oldest animals. NIBSC 21/234 serves as a positive control (open black circle). The grey area is the baseline observed for pre-immune neutralizing activity (dilution 1/5), and serums for the V1 and V2 old animals. Data points beneath this area are below the detection limit.

The specific RBD antibody responses were measured in serums at weeks -4, 2, 4, 6, 8 and 9, with a vaccination at week 0 and 4 and the challenge from week 8 to week 9 (Fig. 1). Pre-existing influenza immunity did not prevent virosome-RBD immunogenicity, serum anti-RBD IgG titers are shown for immunized animals (Fig. 3a), with a pre-immune serum geomean titers (GMT) baseline < 100 that increases to 5,525 at four weeks after the first immunization, and four weeks after the second immunization (week 8) the serum GMT increased to 61,420. Those vaccine-induced serum GMTs at week 4 and 8 were significantly higher (*p* = 0.0095) than the serum GMTs of the control group (GMT around 100). Notably, macaques with the lowest serum titers (animals V1 and V2) were also the two older animals from that vaccinated group (Table 1). In our NHP study, we observed that at week 8, a weak serum IgA response was detected in only two macaques out of six (Fig. 3a), which are animals with high serum IgG titers. Further investigations are required to understand these differences.

**Table 1:** Animal name, age and weight at the start of the study.

Animal name	Animal code	Age (years)	Weight (kg)
Vaccinated group			
R05078	V1	16.03	17.85
R12080	V2	9.18	12.47
R13071	V3	8.22	16.95
R16096	V4	5.02	9.35
R17037	V5	4.24	8.10
R17114	V6	4.16	7.25
	Mean	7.81	12.00
Control group			
R14135	C1	7.09	14.20
R16085	C2	5.07	9.30
R11079	C3	10.17	13.80
R13123	C4	8.15	10.30
	Mean	7.62	11.90

Three of the vaccinated animals also had RBD specific IgG (but no IgA) in the nasal wash (Fig. 3b) and in BAL fluid (Fig. 3c) collected at week 7, while there were none detected in the control group (Fig. 3b). One week after SARS-CoV-2 challenge (week 9), all control animals developed RBD-specific serum IgGs and a modest IgA response (Fig. 3a), while the serum IgG responses decreased in five out of six immunized animals, with only animal V1 showing an increase in IgG levels. Despite the decrease in IgG, there was an increase in RBD specific IgA responses in five out of six immunized animals. Following the challenge, all immunized and control animals showed either an increase or became positive for RBD specific IgG and IgA in the nasal wash (Fig. 3b). After euthanasia, BAL were not collected due to the risk of interference with the histopathology evaluation.

Emerging SARS-CoV-2 variants of concern (VOC) have increased resistance to neutralization by antibodies that reduces the vaccine efficacy^47^, with altered virus transmissibility, pathogenicity, and infection effectiveness^71^. Because the animal protection assesses only the SARS-CoV-2 Wuhan-like strain, it does not predict about the protection toward other VOCs. Therefore, the cross-strain serum neutralizing antibodies (week 8) were evaluated (Fig. 3d), using a validated neutralization assay with live viruses. The determined geomean (GM) neutralizing antibody titers (NT) were the following: Wuhan-like (GM NT 42), Alpha B.1.1. 7 (GM NT 85), Beta B.1.351 (GM NT 16), Delta B.1.617.2 (GM NT 40) and Omicron B.1.1.529 (geomean NT 7). When looking individual animals, the V1 oldest animals with the lowest serum titer (Fig. 3a) has also no neutralizing activity (Fig. 3d), and this impact strongly the calculated mean titers and it is preferable to show the geomean neutralizing titers. The four youngest animals with the highest serum titers (V3 to V6), those serums neutralize equally well the Wuhan, Alpha, Delta but slightly less the Beta strain (Fig. 3d), with no significant difference between the Wuhan and the most distant Delta variant (*p* = 0.750), The Omicron B.1.1.529 was poorly neutralized and this is most likely due to the heavily mutated RBD surface buried in the S down position of that variant^48^.

RBD specific T-cell responses, as measured by IFNγ and IL-4 ELISpot assay, remained below detection level in all immunized animals (not shown). These results contrast with our rat study, immunization with virosome-RBD led to significant levels of T cells producing IFNγ and only low IL-4 responses in the draining lymph nodes (data not shown).

**Figure 4:**
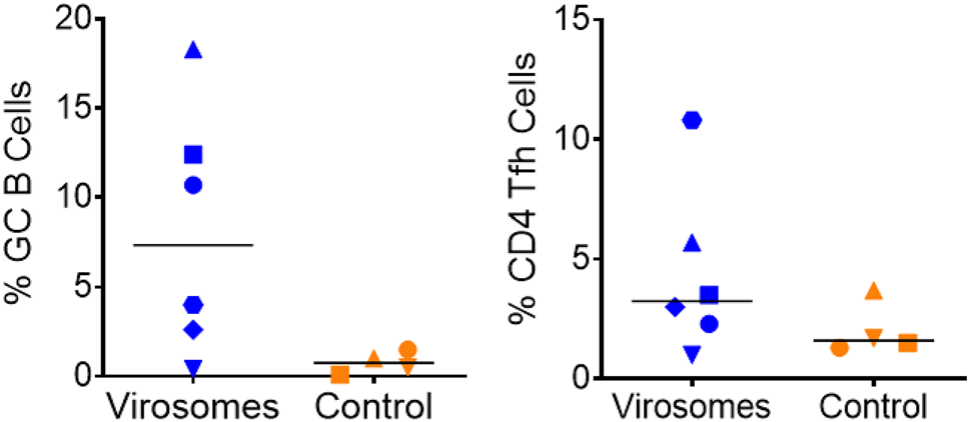
Immunization with Virosomes-RBD induces germinal center B cells in the spleen. The relative percentage of germinal center (GC) B cells over total B cells (left panel) and CD4 + T follicular helper cells (Tfh) over total CD4 + T cells (right panel) in the spleen is shown. Virosomes-RBD vaccinated (blue) and control animals (orange). Median values are indicated by horizontal black lines. Although we observed an increase in the percentage of GC B cells in the spleen at the day of necropsy in the virosomes-RBD group, it does not reach the 0.05 significance threhold (*p* = 0.068), in part because the increase was observed for only 3 out of 6 animals. Tfh cells were not significantly different between the virosomes-RBD vaccine and the control group (*p* = 0.325).

One week after the challenge, the spleen cells were collected, and he percentage of Germinal center (GC) B cells, respective to the total B cell population, was relatively high in five out of six animals from the vaccinated group, as compared to the control group (Fig. 4), but did not reach significance (*p* = 0.068). Higher numbers of spleen follicular helper (Tfh) cells were only observed in 2 out of 6 vaccinated but there is no significant difference between the two groups (*p* = 0.325). Possibly, the five weeks post-vaccination time point might have been suboptimal for evaluating the Tfh cell population. Although the spleen was collected one week after the viral challenge, the infected control group had no or very low detectable GG B cells in the spleen and therefore, the virus production had no significant impact on the GC B cell number. This implies that vaccine-induced immune responses are mainly responsible for the increase of spleen GC B cells.

### Virosomes-RBD vaccine efficacy against SARS-CoV-2 challenge

**Figure 5:**
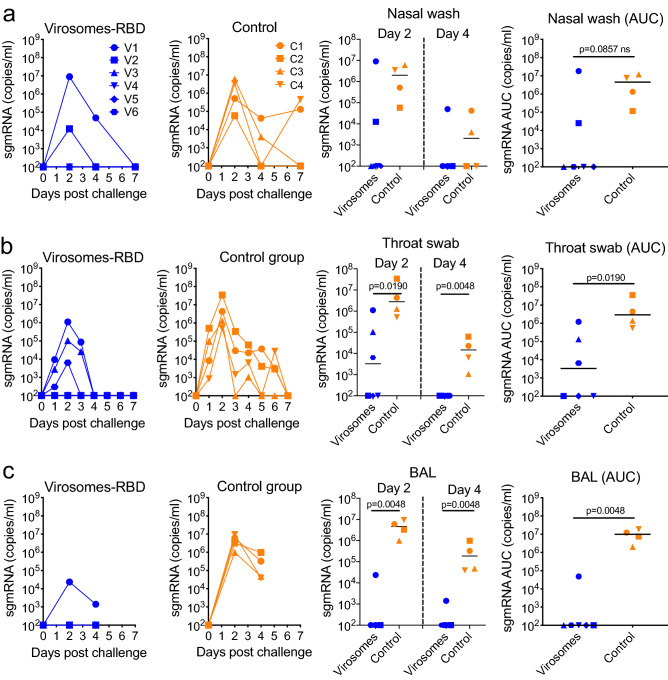
Control of SARS-CoV-2 replication by virosomes-RBD vaccine. SARS-CoV-2 sgRNA load (copies/mL) and area under the curve (AUC) for the sgRNA load (copies/mL) in nasal washes (**a**), throat (**b**) and lung bronchoalveolar lavages (BAL) **(c)** of virosomes-RBD immunized macaques (blue, left panels) and control animals (orange, middle panels) at different days post-challenge. Each animal is represented by a symbol. All data are shown relative to the day of challenge (day 0, day of virus administration) done at day 56 of the study. Panels on the right show statistical evaluation on SARS-CoV-2 sgRNA load in nasal washes on day 2 and 4 (upper right), in the throat (middle right panel) and in BAL fluid on day 2 and 4 (lower right panel). Median values are indicated by horizontal black lines. Significant differences between the groups are indicated in the graph by a horizontal line and *p*-value (Mann–Whitney test).

The virosomal vaccine efficacy for preventing SARS-CoV-2 infection and replication in the upper and lower respiratory tract was daily monitored by quantifying the subgenomic mRNA (sgRNA) by real-time PCR as a marker of replicating virus in nasal washes, the throat (swabs) and the lungs (BAL fluid) (Fig. 5). All control animals became virus positive in the nasal wash, throat and BAL. In contrast, three out of six vaccinated animals had no detectable virus in either nasal wash, throat or BAL. Animal V1 that is the oldest vaccinated animal with the lowest serum RBD antibody titers had the highest virus load in nasal wash and throat, comparable to the virus loads observed in the control group, and this V1 animal was the only vaccinated animal that showed low level virus replication in the BAL (Fig. 5c). Two other animals, V3 and V6, showed modest to low virus replication in the throat and animal V2 developed low virus levels in the nasal wash. For the sgRNA in nasal wash at day 2 and 4 post challenge, there was no significant difference between the vaccine and control group (Fig. 5a, right panel), while there was a significant sgRNA reduction in the throat and BAL of vaccinated animals. In the immunized animals, no viral sgRNA could be detected in any of the processed tissues of the respiratory tract, which included the nasal mucosa, trachea, right and left bronchus and seven lung lobes (data not shown). Instead, three of the four control animals had detectable virus in either one (animal C1) or five (animal C2 and C4) lung lobes, while no virus was detected in the tissues of animal C3 (data not shown). Those data clearly indicate that animals vaccinated with virosomes-RBD were protected against SARS-CoV-2, although the old V1 animal had a lower level of protection.

### Inflammatory cytokine profile and lung histology in macaques exposed to SARS-CoV-2

**Figure 6:**
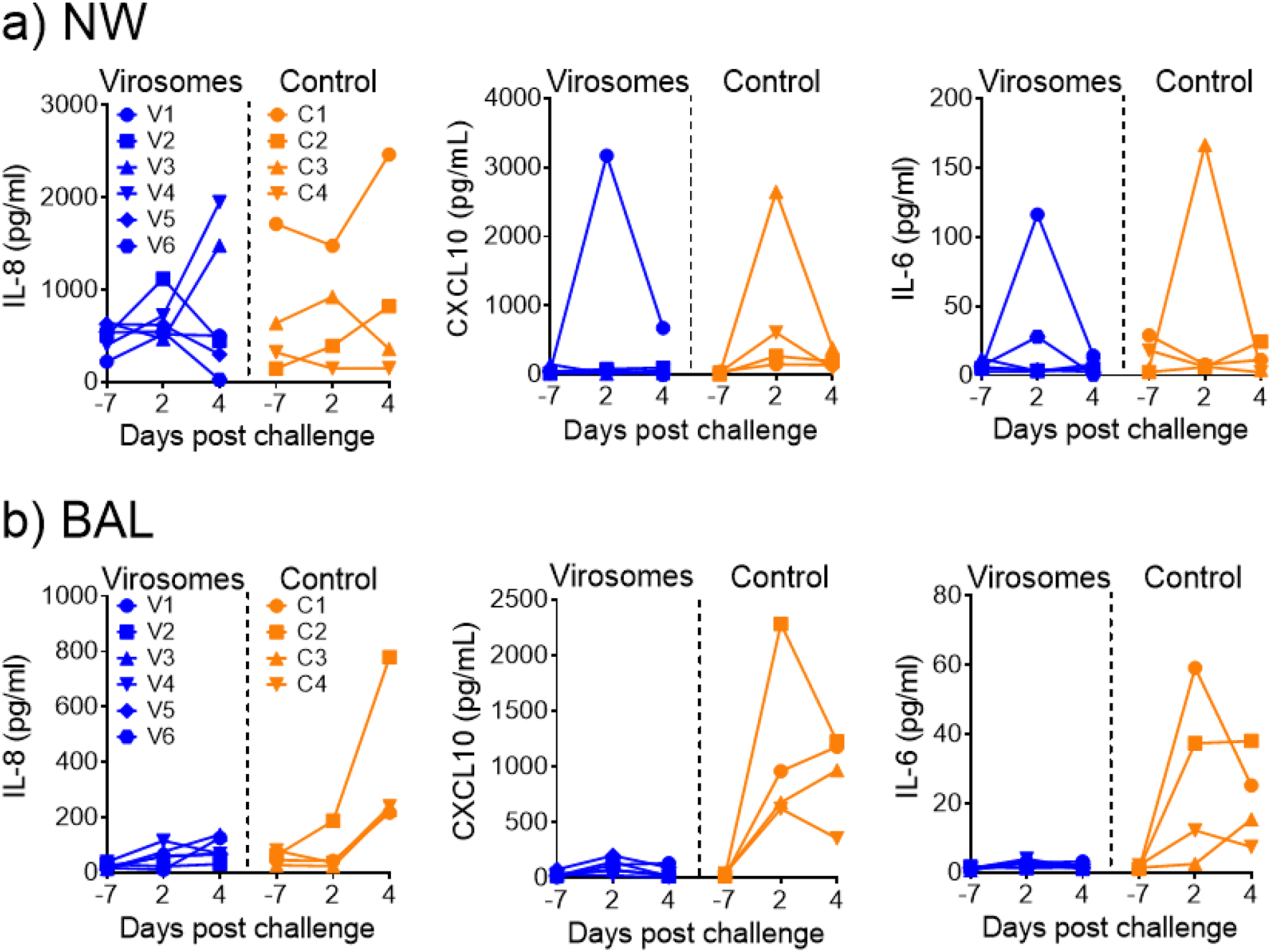
Control of SARS-CoV-2-induced cytokine storm in BAL fluids, but not in nasal washes, after intramuscular virosomes-RBD vaccination. Pro-inflammatory cytokine levels of IL-8, CXCL10 and IL-6 in (**a**) nasal washes (NW) and (**b**) BAL fluid of virosomes-RBD immunized (blue) and control (orange) animals. All data are shown relative to the day of challenge (day 0). Levels in each individual animal (indicated by different symbols) in time are shown. No measurements were performed on day of challenge, but data obtained at day 7 before challenge are shown.

It was previously shown that SARS-CoV-2 infection in humans and in NHPs induces an increase in inflammatory cytokines and chemokines in BAL and serum samples^72–74^ and that COVID-19 vaccines can provide a protective effect against such increase^75–77^. In agreement with these studies, we observed increased levels of IL-8, CXCL-10 and IL-6 in BAL samples of infected control animals, but not in any of the six vaccinated animals (Fig. 6b). In this study, the nasal washes were also analyzed. However only one of the four control animals showed increased levels of these inflammatory cytokines and chemokines, peaking at d4 for IL-8 and at d2 for CXCL10 and IL-6. The control animal C1 that remained strongly virus positive until d7 (Fig. 5a) and animal C4 that showed again an increased production of virus in the nose at d7, were the animals that showed these increased cytokine levels (Fig. 6a). A similar increase was also observed in the vaccinated old animal V1 that also had the highest level of virus replication in the nose. The data suggest that virus replication in the nose and lungs drives the induction of pro-inflammatory cytokines, which is more consistently observed in BAL samples.

**Figure 7:**
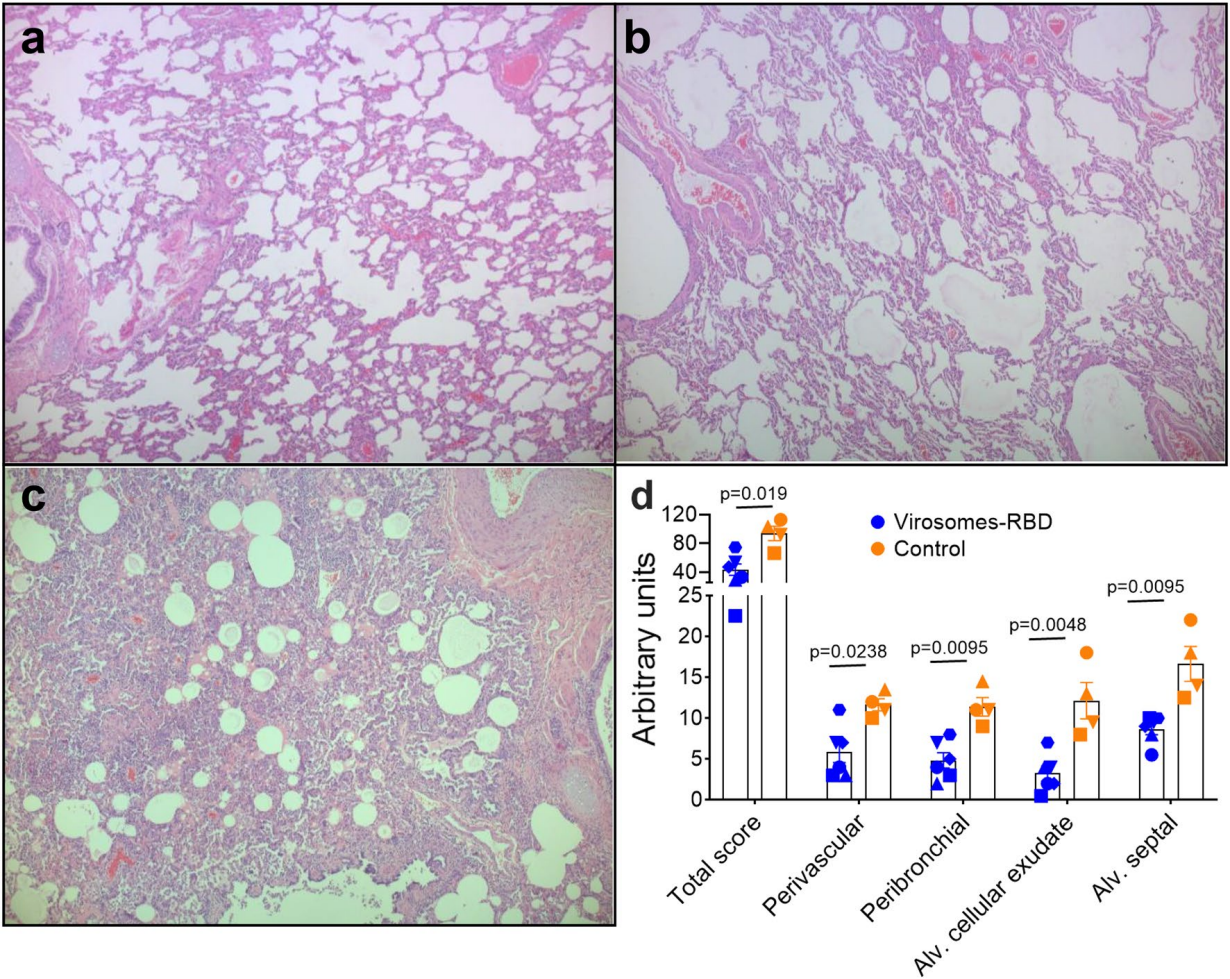
Immunization with virosomes-RBD reduces lung inflammatory pathology. Lung histopathology visualized by light microscopy of hematoxylin and eosin (H&E) tissue staining of lung lobes from representative animals of each group. (**a**) Lung tissue from an uninfected animal showing intact pulmonary structures and lack of inflammation. (**b**) Lung tissue of animal V3 from the virosomes-RBD vaccine group exhibits minimal to mild interstitial infiltrate of inflammatory cells, and rare oedema and fibrin. (**c**) Lung tissue of animal C1 from unvaccinated, infected (control) group with histological features of moderate to severe interstitial inflammation, alveolar cellular infiltrates, abundant oedema and frequent fibrin presence. Image magnification 50x, representative images are shown. (**d**) Lung inflammatory pathology scores for the vaccinated (blue dots) and control group (orange dots). The total pathology score as well as the scores for perivascular inflammatory infiltrates, peribronchiolar inflammatory infiltrates, alveolar cellular exudate, and alveolar septal inflammatory cells are shown for animals immunized with virosomes-RBD (blue symbols) and control animals (orange symbols). Maximum total score is 336 and maximum score per individual parameter is 28. Significant differences between the groups are indicated in the graph by a horizontal line and p-value (Mann–Whitney test).

Euthanasia was planned at day 7, 8 or 9 post SARS-CoV-2 infection, with the manipulation of only two to four animals during each day due to the heavy workload for sample preparation during necropsy. Pulmonary lobes (upper, middle, lower, accessory right and upper, middle, lower left) were processed for histopathological analyses. Microscopic examination showed reduced lung pathology in the immunized animals (Fig. 7b), as compared to the control animals (Fig. 7c), although some residual pathology was still observed in the immunized animals, as compared to healthy uninfected control (Fig. 7a). Quantitative assessment showed in immunized animals a reduction in total inflammatory pathology score as well as reduced levels of perivascular inflammatory infiltrates, peribronchiolar inflammatory infiltrates, alveolar cellular exudate and oedema, and alveolar septal inflammatory cells (Fig. 7d). Histology data have confirmed that vaccinated animals have better preserved lung tissues than control animals, indicating that RBD-induced antibodies have protected from severe SARS-CoV-2 lung pathology.

## Discussion

There is a growing interest for developing COVID-19 vaccines with regular strain adjustment for the S antigen or combining S antigens from different strains for a broader cross-strain antibody repertoire. However, studies have indicated that the various neutralizing regions are not equally accessible or exposed on the S protein of different virus strains, which can impact the breadth of the natural or S-based vaccine induced neutralizing antibodies^78^. For these reasons, a RBD-based vaccine is another attractive alternative vaccine strategy^79^. Most neutralizing antibodies predominantly target the RBD region^15–20^ that also harbors the few highly conserved antibody epitopes shared across other SARS-CoV-2 Beta coronaviruses^44,80–82^. Therefore, the RBD antigen without the surrounding S antigen structure could fully expose all potential neutralizing regions, favouring a focused immune response toward all key RBD sites, including cross-strain neutralizing antibodies to support the development of pan-sarbecovirus vaccines.

Furthermore, most of the human population now harbours low to high levels of natural and/or vaccine-induced immunity toward SARS-CoV-2. In this context, subsequent recall vaccination with boosts may not necessarily require the administration of high antigen and adjuvant dose, as performed during the primary vaccination regimen with two vaccine doses in naive subjects. Therefore, we designed and tested a COVID-19 vaccine with a lower RBD dose (only 15 μg) with a single adjuvant at a very low dose (1 μg), administered intramuscularly to macaques of this NHP study, but other studies are ongoing for evaluating a similar virosome-RBD/3M-052 formulation for the nasal route.

Such virosomal vaccines should retain good immunogenicity due to the presence of repetitive antigen motifs (RBD) displayed at the surface of particles that favors the cross linking of cell surface receptors that improves the immune system activation^55,56^. Therefore, dose sparing effect for both antigen and adjuvant is expected with virosomes as enveloped VLPs displaying RBD with adjuvant bound on the same particle^56^. The absence of free-form adjuvant is expected to prevent systemic circulation and minimize unspecific immune activation, improving the vaccine tolerability and safety profile^83^. When 3M-052 is physically bound to the antigen/particle, it improves the Th1 CD4 + and CD8 + T cell responses, respective to free 3M-052^84^. Therefore, similar observations might be also achieved with virosomes-RBD/3M-052 but this could not be investigated during our NHP study.

Because the tested liquid vaccine is based on the nasal virosomal vaccine with a low dose adjuvant for safety reason, the intramuscular immunization was not expected to elicit a strong humoral RBD specific response. However, if such a vaccine candidate still supports protection in naïve macaques, one can then assume that a similar or stronger immune response would be achieved in NHPs or humans that acquired pre-existing SARS-CoV-2 immunity recently. The low adjuvant dose in our virosomal COVID-19 vaccine candidate is also a main differentiator, respective to most RBD-based subunit vaccines, using the RBD monomeric form^85^, the dimeric RBD^86^ or VLP displaying RBD^77^ that contain a high dose of adjuvant. Excluding alum based adjuvant, higher doses are used with squalene based oil-in water adjuvant^87^, 25 μg of M-Matrix^88^, 500 or 750 μg of each of CpG-alum combination^85^ or 5–10 μg of 3M-052 mixed with alum^89,90^.

The currently tested COVID-19 vaccine based on virosomes-RBD/3M-052 was well tolerated and no safety or tolerability concern arose from the investigations, based on the monitoring of local and systemic effects, animal behavior, clinical chemistry, hematology, and the inflammatory cytokine profile measured in the serums, nasal washes and BALs (IL-1β, IL-6, IL-8, IL-10. IL-12p40, IL-17A, IL-23, IFNβ, IFNγ, TNFα, CXCL10 and CCL2). This excellent tolerance and safety profile is in line with other virosome-based vaccines toward various pathogens previously evaluated in humans^60,61,66,67^.

Our study in male Rhesus macaques showed that a good priming in 5 out 6 animals (Fig. 3a) was observed, with a serum geometric mean titer (GMT) of 6,400, and after the second vaccine administration, a good boost effect is observed in all animals (GMT 97,000). However, the oldest animal V1 (16 years) had a weak antibody response after two immunizations. Regarding the immunogen-induced IgAs (Fig. 3a), the response was generally low respective to the IgG response, which correlates with the relative abundance of IgG over the IgA isotype in the serum, but was detected only in two animals with high serum IgG titers. This may suggest that the detection of IgA is more likely feasible if the vaccine induces high serum titers.

Although most vaccinated animals have only low to intermediate serum titers, we evaluated the macaque serums in microneutralization with life viruses (Fig. 3d), keeping in mind the known correlation between serum titers and serum neutralizing activity. For the four animals (V3-V6) with good serum titers, comparable neutralizations were observed between the Wuhan-like, Alpha B.1.1.7, and also with the most distant Delta B.1.617.2 strain, while it was lower for the Beta B.1.351, with the lowest neutralizing activity toward the Omicron B.1.1.529. The data provide evidence that RBD can induce heterologous protection, but not necessarily toward all variants. The weak neutralization of Omicron is more likely due to poor antibody binding to the RBD of this VOC^44,48^. This suggests that RBD might not be always accessible on variants. For an optimal pan-sarbecovirus vaccines, it might be required, in addition to combine RBD antigens of different strains, to include S2 or other antigen targets that harbor different and complementary conserved epitopes.

Designing a RBD-vaccine triggering antibodies toward most if not all RBD regions might have the best chance to achieve the broadest cross-strain protection, but this may also depend on how the RBD antigen is exposed to the immune system. Our virosomal vaccine displays Wuhan derived RBD with a certain number of random orientations, with the objective to maximize most epitope exposure. Meanwhile, we cannot exclude the possibility that epitopes underneath a given RBD anchored antigen proximal to the virosome membrane are poorly accessible. Ideally, the virosomal RBD vaccine should contain RBDs from two or three most representative VOC for eliciting the broadest protection toward a maximum number of VOC.

All vaccinated animals were protected, with 5 out of 6 animals with no detectable sgRNA in BAL. The oldest vaccinated animal (V1) with the lowest serum titer had a single timepoint (day 2) with low BAL sgRNA. The three youngest animals had also no detectable RNA in nasal washes and throat swabs. Importantly, all vaccinated macaques were protected against severe SARS-CoV-2 lung pathology disease (Fig. 7), as shown by lower inflammatory scores than the control animals. Despite the presence of suboptimal RBD specific serum antibody titers with low serum neutralizing titers in 4 out of 6 animals (V2, V3, V5 and V6), with the oldest animal (V1) having no detectable serum neutralizing antibodies toward the Wuhan-like strain, it was remarkable that all vaccinated animals were protected against the lung pathology. Importantly, all vaccinated animals were also protected against an increase of the pro-inflammatory cytokines IL-8, CXCL-10 and IL-6 in the BALs, while those cytokines were increased in BALs of all infected control animals, which may drive a stronger recruitment of inflammatory cells into the lungs, causing more tissue damages.

Although the limited number of animals doesn’t allow statistical analysis, we noticed that only the youngest macaques (V4 to V6, < 6 years of age during immunization) had detectable nasal and BAL IgGs (Fig. 3b,c). The nasal tissue is the primary SARS-CoV-2 replication site from where it spreads. The presence of nasal antibodies in the three younger animals (V4-V6) could also have contributed to neutralize a fraction of the SARS-CoV-2 inoculum administered during the challenge, and further prevented the early virus infection event in the nasopharynx, explaining the absence or very low levels of detectable sgRNA in the nose and throat of those animals (Fig. 5). It remains to be determined if such nasal antibodies could also reduce the virus transmission from vaccinated to naïve animals. Considering the importance of the mucosal antibody front line defense during early virus acquisition and infection in the upper and lower respiratory tract, we can postulate that the younger vaccinated population is more efficiently protected by intramuscular vaccines than the older vaccinated population, which could be potentially due to better systemic IgG migration to the respiratory tract.

Immunosenescence or the immune system aging alters the function and quality of immune organs and immune cells, and consequently, the immunization of older individuals is generally associated with an immune response of a lower quantity and quality that contributes to a lower vaccine protection^91^. Therefore, detecting more SARS-CoV-2 sgRNA in the nasal, throat and BAL in the older animals might not be surprising, while in younger animal with a stronger immunity there are no detectable viruses. We should point out here that intramuscular vaccines are known to poorly induce good mucosal immunity. Therefore, achieving sterile immunity in the nose, throat and lungs is unlikely achievable. Some viruses could reach the upper and lower respiratory tracts and cause mild lung tissue inflammation under control and prevent disease complications (Fig. 7), for this reason, some people may prefer the term partial protection.

There are evidence that SARS-CoV-2 specific CD4 + and CD8 + T cell responses may contribute to virus clearance and milder disase^92^. Interestingly, protein mutations affecting antibody epitopes have a limited impact on epitopes recognized by the memory T cells, as the overall T cell reactivty is maintained toward most defined T cell peptides in vaccinated or SARS-CoV-2 exposed subjects^93^. Therefore, the presence of CD4 + and CD8 + T cells induced during natural infection and/or vaccination could indeed contribute to protection against future VOC.

The proposed virosomes-RBD vaccine is mainly intended for the induction of protective antibodies but virosomes can also induce CD8 T cells^51–53^. However, the frequency of induced CD8 T cell is expected to be low, particularly with a low RBD dose, as compared to viral vectors and mRNA that support de novo protein synthesis with efficient loading of major histocompatibility complex class I antigens with antigen-derived peptides. Furthermore, RBD is a small antigen (25 kDa) with a more limited set of potential peptides for eliciting a strong CD8 T cell response, as compared to a bigger S protein (180 kDa). These aspects may explain the difficulty to detect RBD specific T cell responses in our study. We cannot exclude the possibility that with a higher RBD dose, beside the improvement of the B cell response, also a higher frequency of CD8 T cells could be elicited in SARS-CoV-2 naïve subjects or boost the pre-existing RBD specific memory T cells established after vaccination and/or natural SARS-CoV-2 antigen exposure.

With the complex logistic related to the vaccine freezing conditions during distribution and storage, it is encouraged to develop vaccines that are stored refrigerated (+ 2–8 °C). For this vaccine efficacy study on NHP, we used refrigerated liquid virosomes for intramuscular administration. Recently, we optimized a thermostable powder formulation with virosomes-RBD/3M-052 for nasal administration that is under currently investigation, as an alternative to the intramuscular route. Such nasal formulation will allow cold chain independent transport and is expected to efficiently elicit and boost protective mucosal IgG and IgA antibodies in the nasal and lung tissues.

## Material and methods

### RBD antigen

The recombinant SARS-CoV-2 RBD219-N1C1 protein was designed and produced originally at Baylor College of Medicine (Texas, USA), using previously published methods^49,94^. The RBD219-N1C1 construct encodes a monomeric RBD protein of 218 amino acids (332–549) produced in *Pichia pastoris*, with the deletion of the asparagine 331 (N331) and the mutation of the last cysteine residue by an alanine substitution (C525A).

### Virosome formulation

For this immunogenicity study, RBD219-N1C1 protein was conjugated on a limited amount of lysine residues (≤ 4) to a reactive phospholipid (1,2-Dipalmitoyl-sn-glycero-3-phosphoethanolamine-N-(succinimidyloxy-glutaryl)-L-α-phosphatidylethanolamine, NOF Europe, Belgium). Virosomes with lipidated RBD integrated into their lipid bilayer were prepared as described earlier^69,95^.

Briefly, β-propiolactone-inactivated influenza virus A/Brisbane/59/2007 H1N1 (Seqirus, Australia) was solubilized in 100 mM 1,2 dihexanoyl-sn-glycero-3-phosphatidylcholine (DCPC) (Avanti Polar Lipids, Alabaster, AL, USA) in HN buffer (5 mM HEPES, 142.5 mM NaCl, pH 7.4) and the viral nucleocapsid was removed by high-speed centrifugation. To the supernatant, solubilized synthetic 1,2 dioleyl-sn-glycero-3-phosphatidylcholine (DOPC, Merck & Cie, Schaffhausen, Switzerland) in 100 mM DCPC, HN buffer was added. DCPC was then removed by dialysis in a gamma-irradiated slide-A-lyzer cassette (10 kDa cut-off; Thermo Scientific, Geel, Belgium) against HN buffer. The resulting pure and homogenous virosomes were stored at + 2–8 °C until further use. Adjuvant was inserted into the virosomal membrane by post-insertion. Briefly, 3M-052 (3M, St. Paul, MN, USA) was dissolved in ultrapure ethanol, and a small quantity of this adjuvant was rapidly mixed with the virosomes and incubated for 30 min at RT before long-term storage at + 2–8 °C.

RBD-virosomes were characterized as described^69^ for their contents, RBD and 3M-052 by UPLC, and influenza HA by ELISA. Particle size and homogeneity was determined by nanoparticle tracking (NTA). Antigen and adjuvant integration was confirmed by sucrose density gradient analysis followed by UPLC. RBD coupling and epitope integrity on conjugated RBD was demonstrated by a sandwich ELISA in which antibodies to the virosomal hemagglutinin were used to coat ELISA plates. After blocking, plates were incubated with virosomes and the presence of native RBD on the virosomes was verified by using specific conformation-dependent antibodies to RBD^78^. The final virosomes were 0.22 µm sterile filtered and aliquoted into glass vials. Two separated glass vials were prepared, one for each day of immunization (d0 and d28), and each glass vial for intramuscular administration was supplied in HN buffer, containing 30 μg/mL of RBD and 2 μg/mL of 3M-052 as TLR7/8 agonist.

### Non-human primate study

The study was performed with 10 adult healthy male rhesus macaques (*Macaca mulatta*) randomly distributed into one vaccine treated group with 6 animals (V1-V6), and one untreated group (non-vaccinated) serving as control group with 4 animals (C1-C4). Age and weight of each animal is specified in Table 1. Enrolled animals were free of fecal pathogens and showed normal clinical chemistry and hematology values and did not have serum antibodies toward simian T-cell leukemia virus (STLV), to simian retrovirus (SRV), or to the RBD of the Spike protein of SARS-CoV-2, as tested by ELISA. Animals were housed in pairs with a compatible cage mate in ABSL3 facilities at the BPRC. The macaques were offered a daily diet which was optimized for rhesus macaques. Different enrichment items (toys, extra food puzzles) were offered daily. Drinking water was available ad libitum. At the start of the study (at time of influenza vaccination), the age of the animals ranged from 4.15 to 16.03 years (mean age 7.74 years), and they weighed between 7.02 and 18.27 kg (mean weight 11.79 kg). Animal anesthesia was performed according to the following procedures: (a) For blood sampling and intramuscular immunizations, we used Ketamin 5 mg/kg with Medetomidine 0.05 mg/kg (administered intramuscularly); (b) For BAL sampling and nasal wash, we used Ketamin 10 mg/kg with Medetomidine 0.05 mg/kg (administered intramuscularly). To help for faster recovery after anesthesia, animals received intramuscularly Atipamezol at 0.25 mg/kg. For euthanasia, animals received first intramuscularly Ketamin at 12 mg/kg with Medetomidine 0.05 mg/kg, then Pentobarbital at 70 mg/kg administered intravenously.

The study protocol was reviewed and approved by the Dutch “Centrale Commissie Dierproeven” (AVD5020020209404-2) according to Dutch law, article 10a of the “Wet op de Dierproeven” and BPRC’s Animal Welfare Body.

All methods described into the protocol were performed according to Dutch guideline and approved by the Ethics Committee for the Biomedical Primate Research Centre (Rijswijk, Netherlands). The study is reported in accordance with ARRIVE guidelines.

### Immunization schedule and SARS-CoV-2 challenge

Four weeks prior to vaccination, all animals (including controls) were primed intramuscularly with 0.5 mL of whole inactivated Influenza virus, containing 15 µg HA (A/Brisbane/59/2007 H1N1, solution at 30 µg HA/mL). Then, animals V1-V6 were immunized intramuscularly twice at day 0 and day 28 with 0.5 mL of adjuvanted virosomes-RBD (15 μg of RBD and 1 μg of 3M-052 adjuvant). Local reactions at the immunization sites were recorded on days 1, 2, 3, and 4 following each immunization by Draize scoring. Clinical chemistry and hematology measurements were performed to monitor systemic adverse effects. Two weeks post each vaccination, blood was drawn to monitor B and T-cell immune responses. Four weeks post final vaccination, at day 56 (week 8) all animals were exposed to 1 × 10^5^ TCID_50_ SARS-CoV-2 (isolate BetaCoV/German/BavPat1/2020 p.4) that is like the Wuhan strain but containing the D614G mutation (obtained from the European Virus Archive, Charitéplatz 1, Berlin, Germany). Virus challenge was performed through the combined intranasal (0.25 mL per nostril) and intratracheal (1.5 mL) route. Animals were monitored for 7–9 days post challenge during which samples were collected: Nasal washes, throat swabs and bronchoalveolar lavages were analyzed for the presence of subgenomic RNA (sgRNA) representing replicative viruses, and animals were sacrificed, and lung histopathology was studied (see Fig. 1).

### ELISA

Individual serum samples obtained from rhesus macaques before and at two weeks after each immunization, at time of challenge and one week post SARS-CoV-2 challenge, were tested for the presence of binding IgG against SARS-CoV-2 RBD proteins using an ELISA, as previously described^77^. IgA antibodies were measured using a commercial monkey IgA ELISA kit (Mabtech, Nacka Strand, Sweden) according to manufacturer’s instruction. Additionally, nasal washes taken three weeks after the second immunization and post challenge and BAL fluid from three weeks after the second immunization were tested for the presence of IgG and IgA antibodies. Half area plates were used, that were coated overnight at 4 °C with 1 µg/mL recombinant RBD Spike protein of SARS-CoV-2 (ExpreS2ion Biotechnologies, Horsholm, Denmark), 50 µL per well. For IgG antibody detection the plates were blocked with PBS/3%BSA w/v (blocking solution) for 2 h at 37 °C. Serum, nasal wash or BAL samples were applied in two step serial diluted starting from 1:100 for serum, 1:5 for nasal washes and 1:10 for BAL fluid in PBS/1%BSA/0.05% Tween-20 and incubated 1 h at 37 °C. Bound anti-RBD antibodies were detected using GAHu-IgG (H + L)-HRP (1:1250 in 1% BSA/PBS). The reaction was developed with TMB substrate (50 µL/well for 20 min at RT in the dark) and stopped with 50 µL/well Stop solution (2N H_2_SO_4_). The absorbance was measured at 450 nm.

### SARS-CoV-2 neutralization

The presence of serum neutralizing activity was outsourced to VisMederi (Siena, Italy)^96,97^, using a validated method with optimized conditions for each tested strain for detecting the cytopathic effect. Briefly, the micro neutralization assays were performed with Vero E6 cell line (from American Type Culture Collection) and live virus SARS-CoV-2 produced by VisMederi: Original strain 2019 nCOV ITALY/INMI1 similar to Wuhan, Alpha variant B.1.1.7 from the United Kindom, Beta variant B.1.351 from South Africa, the Delta variant B.1.617.2 from India and the Omicron B.1.1.529 from South Africa. As positive serum control (opened circle), we used the commercially available NIBSC human serum pool 21/234 containing high neutralizing antibody titer toward the SARS-CoV-2 original Wuhan-like strain. All macaque serums were heat inactivated at 56 °C for 30 min prior use. The neutralization titer (NT) is the reciprocal of the highest serum sample dilution that protects at least 50% of cells from cytopathic effect (NT50%).

### Cytokine and chemokine detection

Nasal wash samples and lung brochoalveolar lavages (BAL) were tested for the presence of cytokines and chemokines using the LEGENDPLEX assay (Biolegend LEGENDplexTM NHP Inflammation Panel (13-plex, article no. 740332), according to the manufacturer’s instruction with some minor modifications. Provided by the manufacturer are incubation plates, assay buffer, matrix solution, bead solution, wash buffer and standards for each cytokine. Incubation plates were first pre-wet by adding 100 µL/well wash buffer. After removal of wash buffer, 15 µL of matrix-B followed by 15 µL of pre-diluted standard was added to the appropriate wells. For the test samples, first 15 µL of assay buffer was pipetted into the well, followed by 15 µL of undiluted sample. The standards were in duplicates, samples were tested singly. 15 µL of bead solution was added to all wells. After 2 h incubation at RT in a shaker, wells were washed twice with 100 µl/well wash buffer, 15 µL/well of detection antibody was added and plates were additionally incubated for 1 h at RT. Subsequently, 15 µL/well of Streptavidin-Phycoerhythrin was added, plates were incubated for 30 min. at RT, then washed twice with 100 µL/well wash buffer and beads were finally resuspended in 100 wash buffer/well. Samples were transferred to a 96 well plate and measured on a Aurora FACS machine (Cytek, Fremont, CA, USA) and analyzed by using company software. The following cytokines were measured: IL-1β, IL-6, IL-10, IL-12p40, IL-17A, IL-23, GM-CSF, CXCL10 (IP-10), CCL2 (MCP-1), CXCL8 (IL-8), IFNβ, TNFα, and IFN-γ.

### Quantification of subgenomic mRNA

Tracheal and nasal swabs, as well as BAL samples were analyzed for the presence of sgRNA of SARS-CoV-2 E gene using a quantitative real-time PCR as previously described^75,98,99^. Viral RNA was isolated using a QIAamp Viral RNA Mini kit (Qiagen Benelux BV, Venlo, The Netherlands) following the manufacturer's instructions. The RT-qPCR assay was carried out using the Brilliant II QRT-PCR Core Reagent Kit, 1-Step kit (Agilent Technologies BV, Amstelveen, The Netherlands), according to the instructions provided by the manufacturer in a 25 mL volume with final concentrations of 600 nM for both primers, 200 nM for the probe, and 5 nM MgCl_2_, using 10 mL RNA, extracted from 140 mL sample volume. RNA was reverse transcribed for 30 min at 50 °C. Then, after 10 min incubation at 95 °C, the cDNA was amplified for 45 cycles, consisting of 30 s denaturation at 95 °C, followed by a 1-min annealing-extension step at 60 °C. All the reactions were carried out with an iQ5 Multicolor Real-Time PCR Detection System (Bio-Rad Laboratories BV, Veenendaal, The Netherlands). The amount of RNA was quantified based on a standard curve made via in vitro transcription of a synthetic target sequence.

### FACS analysis

FACS analysis was performed on single cell suspensions that had been prepared from spleen obtained at autopsy. Mononuclear cells were isolated by lymphocyte separation medium (LSM) density gradient centrifugation (Organon-Teknica). Cells at the interface were collected and stored in liquid nitrogen. Thawed cells were first incubated with live/dead blue dead cell stain kit (Molecular Probes, cat. no. L23105). After 20 min. incubation, the cells were washed and then incubated with a mAb mixture containing: CD21^BUV563^ (BD bioscience, cat 741,362), CD27^BUV661^ (BD, cat 741,609), CD71^B480^ (BD, cat 746,247), CD95^BV711^(biolegend, cat 305,644), CD38^FITC^ (Stem cell technologies, cat 10,415), goat anti-human IgD^Alexa555^ (Southern Biotechnology Inc, cat 2030–32), CD297^BV785^ (PD1) (biolegend, cat 329,930), CD185^PE^ (CXCR5) (Ebioscience, cat 12–9185-42), IgM^PerCP-Cy5.5^ (BD, cat 561,285), IgG^APC^ (BD, cat 550,931), CD3^Alexa700^ (BD, cat 641,414) diluted in Brilliant Stain Buffer Plus (BD, cat 566,349). After 30 min. incubation at 4 °C in the dark, the cells were washed and fixed overnight at 4 °C in 2% paraformaldehyde solution in PBS. Flow cytometry was performed on an Aurora FACS machine using company software (Cytek, Fremont, CA, USA). For each tube the maximum number of events were recorded. Germinal center (GC) B cells and T follicular helper (Tfh) cells were analyzed by selecting cells within the lymphocyte gate, and then the singlets by using the forward scatter area plotted against the forward scatter height. Subsequently dead cells were excluded and GC were identified as: CD3^neg^/CD20^pos^/CD38^neg^/CD71^pos^ cells, while Tfh were identified as CD3^pos^/CD4^pos^CD279 (PD1)^bright^/CD185 (CXCR5)^pos^ as descrined^77,100^. The cytometry gating strategy is described in the Supplementary section (Fig. S2).

### Lung histophathology

The histopathology scoring on the lung sections was performed by a pathologist blinded to group allocation for having unbiased scores. Tissue samples from all pulmonary lobes, the upper respiratory tract (nasal mucosa, oro/nasopharynx, trachea, left and right bronchus), heart, kidney, liver, ileum and colon were collected for histopathology and preserved by immersion in 10% neutral-buffered formalin for 72 to 96 h. Specimens for microscopic examination were processed, embedded in paraffin and sections of 4 µm were stained with hematoxylin and eosin (H&E). The lesions were quantified as follows: 0 = no lesions, 1 = minimal, 2 = mild, 3 = moderate, 4 = severe, using a pulmonary histopathology scoring system for SARS-CoV-2 infection in macaques as described^75,99^.

### Statistical analysis

Differences between groups were calculated by using the Mann–Whitney test. A two-sided α level of 0.05 was used to determine significance. For the neutralization assay, groups were compared by using the non-parametric Wilcoxon matched pair signed rank test.

### Ethics statement

The animal study was reviewed and approved by the Dutch “Centrale Commissie Dierproeven” or CCD (project code AVD5020020209404-2) according to Dutch law, article 10a of the “Wet op de Dierproeven,” and BPRC’s Animal Welfare Body (IvD).

## Supplementary Information

Supplementary Information.

## Data Availability

The original contributions presented in the study are included in the article material. Under reasonable request, the immunological datasets generated during this study are available from the corresponding author on reasonable request.

## Abbreviations

ACE2: Angiotensin-converting enzyme 2
BAL: Bronchoalveolar lavage
BSA: Bovine serum albumin
COVID-19: Coronavirus disease of 2019
d: Day
DLS: Dynamic light scattering
eVLP: Enveloped virus-like particle
ELISA: Enzyme-linked immunosorbent assay
FcRn: Neonatal Fc receptor
GC: Germinal center
GMT: Geomean titer
HA: Hemagglutinin
H_2_SO_4_: Sulfuric acid
i.m.: Intramuscular
IgG: Immunoglobulin G
IgA: Immunoglobulin A
mAb: Monoclonal antibody
NHP: Non-human primate
NT: Neutralizing titer
NTA: Nanoparticle tracking analysis
PBS: Phosphate buffer saline
PDI: Polydispersity index
RBD: Receptor binding domain
RNA: Ribonucleic acid
RT: Room temperature
TCID_50_: 50% Tissue culture infectious dose
TLR7/8: Toll-like receptor 7 and 8
SARS-CoV-2: Severe acute respiratory syndrome coronavirus 2
S: Spike envelope protein
sgRNA: Subgenomic ribonucleic acid
VOC: Variant of concern
Tfh: T follicular helper cell
UPLC: Ultra Performance Liquid Chromatography
WHO: World Health Organization

## References

[1] Li Q (2020). Early transmission dynamics in Wuhan, China, of novel coronavirus-infected pneumonia. N. Engl. J. Med..

[2] Zhou P (2020). A pneumonia outbreak associated with a new coronavirus of probable bat origin. Nature.

[3] Wu P (2020). Real-time tentative assessment of the epidemiological characteristics of novel coronavirus infections in Wuhan, China, as at 22 January 2020. Euro. Surveill..

[4] Morawska L, Cao J (2020). Airborne transmission of SARS-CoV-2: The world should face the reality. Environ. Int..

[5] Zhang R, Li Y, Zhang AL, Wang Y, Molina MJ (2020). Identifying airborne transmission as the dominant route for the spread of COVID-19. Proc. Natl. Acad. Sci. U.S.A..

[6] Helmy YA (2020). The COVID-19 pandemic: A comprehensive review of taxonomy, genetics, epidemiology, diagnosis, treatment, and control. J. Clin. Med..

[7] Stefanelli P (2020). Whole genome and phylogenetic analysis of two SARS-CoV-2 strains isolated in Italy in January and February 2020: Additional clues on multiple introductions and further circulation in Europe. Euro. Surveill..

[8] Ahn JH (2021). Nasal ciliated cells are primary targets for SARS-CoV-2 replication in the early stage of COVID-19. J. Clin. Investig..

[9] Balcom EF, Nath A, Power C (2021). Acute and chronic neurological disorders in COVID-19: Potential mechanisms of disease. Brain.

[10] Tai W (2020). Characterization of the receptor-binding domain (RBD) of 2019 novel coronavirus: Implication for development of RBD protein as a viral attachment inhibitor and vaccine. Cell. Mol. Immunol..

[11] Wrapp D (2020). Cryo-EM structure of the 2019-nCoV spike in the prefusion conformation. Science.

[12] Lan J (2020). Structure of the SARS-CoV-2 spike receptor-binding domain bound to the ACE2 receptor. Nature.

[13] Hoffmann M (2020). SARS-CoV-2 cell entry depends on ACE2 and TMPRSS2 and is blocked by a clinically proven protease inhibitor. Cell.

[14] Li MY, Li L, Zhang Y, Wang XS (2020). Expression of the SARS-CoV-2 cell receptor gene ACE2 in a wide variety of human tissues. Infect. Dis. Poverty.

[15] Min L, Sun Q (2021). Antibodies and vaccines target RBD of SARS-CoV-2. Front. Mol. Biosci..

[16] Kumar S, Chandele A, Sharma A (2021). Current status of therapeutic monoclonal antibodies against SARS-CoV-2. PLoS Pathog..

[17] Wang X (2022). A potent human monoclonal antibody with pan-neutralizing activities directly dislocates S trimer of SARS-CoV-2 through binding both up and down forms of RBD. Signal Transduct. Target Ther..

[18] Brouwer PJM (2020). Potent neutralizing antibodies from COVID-19 patients define multiple targets of vulnerability. Science.

[19] Li T (2021). Uncovering a conserved vulnerability site in SARS-CoV-2 by a human antibody. EMBO Mol. Med..

[20] Zost SJ (2020). Rapid isolation and profiling of a diverse panel of human monoclonal antibodies targeting the SARS-CoV-2 spike protein. Nat. Med..

[21] Spiekermann GM (2002). Receptor-mediated immunoglobulin G transport across mucosal barriers in adult life: Functional expression of FcRn in the mammalian lung. J. Exp. Med..

[22] Peter HH (2020). Targeting FcRn for immunomodulation: Benefits, risks, and practical considerations. J. Allergy Clin. Immunol..

[23] Alturki SO (2020). The 2020 pandemic: Current SARS-CoV-2 vaccine development. Front. Immunol..

[24] Fiolet T, Kherabi Y, MacDonald CJ, Ghosn J, Peiffer-Smadja N (2022). Comparing COVID-19 vaccines for their characteristics, efficacy and effectiveness against SARS-CoV-2 and variants of concern: A narrative review. Clin. Microbiol. Infect.

[25] Kaur SP, Gupta V (2020). COVID-19 Vaccine: A comprehensive status report. Virus Res..

[26] Khan WH (2021). COVID-19 pandemic and vaccines update on challenges and resolutions. Front. Cell. Infect. Microbiol..

[27] Krammer F (2020). SARS-CoV-2 vaccines in development. Nature.

[28] Lund FE, Randall TD (2021). Scent of a vaccine. Science.

[29] Maharjan PM, Choe S (2021). Plant-based COVID-19 vaccines: Current status, design, and development strategies of candidate vaccines. Vaccines.

[30] Martinez-Flores D (2021). SARS-CoV-2 vaccines based on the spike glycoprotein and implications of new viral variants. Front. Immunol..

[31] Rinoldi C (2021). Nanotechnology-assisted RNA delivery: From nucleic acid therapeutics to COVID-19 vaccines. Small Methods.

[32] Schoenmaker L (2021). mRNA-lipid nanoparticle COVID-19 vaccines: Structure and stability. Int. J. Pharm..

[33] Tenchov R, Bird R, Curtze AE, Zhou Q (2021). Lipid nanoparticles horizontal line from liposomes to mRNA vaccine delivery, a landscape of research diversity and advancement. ACS Nano.

[34] Vu MN, Kelly HG, Kent SJ, Wheatley AK (2021). Current and future nanoparticle vaccines for COVID-19. EBioMedicine.

[35] Sughayer MA (2022). Comparison of the effectiveness and duration of anti-RBD SARS-CoV-2 IgG antibody response between different types of vaccines: Implications for vaccine strategies. Vaccine.

[36] Zhang Z (2022). Humoral and cellular immune memory to four COVID-19 vaccines. Cell.

[37] Goldberg Y (2022). Protection and waning of natural and hybrid immunity to SARS-CoV-2. N. Engl. J. Med..

[38] Hamady A, Lee J, Loboda ZA (2022). Waning antibody responses in COVID-19: What can we learn from the analysis of other coronaviruses?. Infection.

[39] Lauring AS, Andino R (2010). Quasispecies theory and the behavior of RNA viruses. PLoS Pathog..

[40] Duffy S (2018). Why are RNA virus mutation rates so damn high?. PLoS Biol..

[41] Kim JS (2020). Genome-wide identification and characterization of point mutations in the SARS-CoV-2 genome. Osong. Public Health Res. Perspect..

[42] Li Q (2020). The impact of mutations in SARS-CoV-2 spike on viral infectivity and antigenicity. Cell.

[43] Greaney AJ (2021). Complete mapping of mutations to the SARS-CoV-2 spike receptor-binding domain that escape antibody recognition. Cell Host Microbe.

[44] Hastie KM (2021). Defining variant-resistant epitopes targeted by SARS-CoV-2 antibodies: A global consortium study. Science.

[45] Guruprasad L (2021). Human SARS CoV-2 spike protein mutations. Proteins.

[46] Thakur S (2022). SARS-CoV-2 mutations and their impact on diagnostics, therapeutics and vaccines. Front. Med..

[47] Garcia-Beltran WF (2021). Multiple SARS-CoV-2 variants escape neutralization by vaccine-induced humoral immunity. Cell.

[48] Zhao Z (2022). Omicron SARS-CoV-2 mutations stabilize spike up-RBD conformation and lead to a non-RBM-binding monoclonal antibody escape. Nat. Commun..

[49] Chen WH (2021). Genetic modification to design a stable yeast-expressed recombinant SARS-CoV-2 receptor binding domain as a COVID-19 vaccine candidate. Biochim. Biophys. Acta (BBA) Gen. Sub.j.

[50] Pollet J (2021). SARS-CoV-2 RBD219-N1C1: A yeast-expressed SARS-CoV-2 recombinant receptor-binding domain candidate vaccine stimulates virus neutralizing antibodies and T-cell immunity in mice. Hum. Vaccin. Immunother..

[51] Moser C (2007). Influenza virosomes as a combined vaccine carrier and adjuvant system for prophylactic and therapeutic immunizations. Expert Rev. Vaccines.

[52] Moser C, Amacker M, Zurbriggen R (2011). Influenza virosomes as a vaccine adjuvant and carrier system. Expert Rev. Vaccines.

[53] Moser C, Muller M, Kaeser MD, Weydemann U, Amacker M (2013). Influenza virosomes as vaccine adjuvant and carrier system. Expert Rev. Vaccines.

[54] Asadi K, Gholami A (2021). Virosome-based nanovaccines; a promising bioinspiration and biomimetic approach for preventing viral diseases: A review. Int. J. Biol. Macromol..

[55] Kushnir N, Streatfield SJ, Yusibov V (2012). Virus-like particles as a highly efficient vaccine platform: Diversity of targets and production systems and advances in clinical development. Vaccine.

[56] Cimica V, Galarza JM (2017). Adjuvant formulations for virus-like particle (VLP) based vaccines. Clin. Immunol..

[57] Bovier PA (2008). Epaxal: A virosomal vaccine to prevent hepatitis A infection. Expert Rev. Vaccines.

[58] Gluck R (1992). Immunopotentiating reconstituted influenza virus virosome vaccine delivery system for immunization against hepatitis A. J. Clin. Investig..

[59] Bovier PA (2008). Recent advances with a virosomal hepatitis A vaccine. Expert. Opin. Biol. Ther..

[60] Chappuis F (2017). Immunogenicity and estimation of antibody persistence following vaccination with an inactivated virosomal hepatitis A vaccine in adults: A 20-year follow-up study. Vaccine.

[61] Herzog C (2009). Eleven years of Inflexal V-a virosomal adjuvanted influenza vaccine. Vaccine.

[62] Gasparini R, Lai P (2010). Utility of virosomal adjuvated influenza vaccines: a review of the literature. J. Prev. Med. Hyg..

[63] de Bruijn IA, Nauta J, Gerez L, Palache AM (2004). Virosomal influenza vaccine: A safe and effective influenza vaccine with high efficacy in elderly and subjects with low pre-vaccination antibody titers. Virus Res..

[64] Esposito S (2008). Safe administration of an inactivated virosomal adjuvanted influenza vaccine in asthmatic children with egg allergy. Vaccine.

[65] Schaad UB (2000). Comparison of immunogenicity and safety of a virosome influenza vaccine with those of a subunit influenza vaccine in pediatric patients with cystic fibrosis. Antimicrob. Agents Chemother..

[66] Leroux-Roels G (2013). Randomized phase I: Safety, immunogenicity and mucosal antiviral activity in young healthy women vaccinated with HIV-1 Gp41 P1 peptide on virosomes. PloS One.

[67] Genton B (2007). A randomized placebo-controlled phase Ia malaria vaccine trial of two virosome-formulated synthetic peptides in healthy adult volunteers. PloS One.

[68] Cech PG (2011). Virosome-formulated Plasmodium falciparum AMA-1 & CSP derived peptides as malaria vaccine: Randomized phase 1b trial in semi-immune adults & children. PloS One.

[69] Amacker M (2020). New GMP manufacturing processes to obtain thermostable HIV-1 gp41 virosomes under solid forms for various mucosal vaccination routes. NPJ Vaccines.

[70] Dowling DJ (2018). Recent advances in the discovery and delivery of TLR7/8 agonists as vaccine adjuvants. Immunohorizons.

[71] Hadj Hassine I (2022). Covid-19 vaccines and variants of concern: A review. Rev. Med. Virol..

[72] Blanco-Melo D (2020). Imbalanced host response to SARS-CoV-2 drives development of COVID-19. Cell.

[73] Del Valle DM (2020). An inflammatory cytokine signature predicts COVID-19 severity and survival. Nat. Med..

[74] Lu S (2020). Comparison of nonhuman primates identified the suitable model for COVID-19. Signal Transduct. Target Ther..

[75] Mooij P (2022). Poxvirus MVA expressing SARS-CoV-2 S protein induces robust immunity and protects rhesus macaques from SARS-CoV-2. Front. Immunol..

[76] Routhu NK (2021). A modified vaccinia Ankara vector-based vaccine protects macaques from SARS-CoV-2 infection, immune pathology, and dysfunction in the lungs. Immunity.

[77] Volkmann A (2022). A Capsid virus-like particle-based SARS-CoV-2 vaccine induces high levels of antibodies and protects rhesus macaques. Front. Immunol..

[78] van der Velden YU (2022). A SARS-CoV-2 Wuhan spike virosome vaccine induces superior neutralization breadth compared to one using the Beta spike. Sci. Rep..

[79] Kleanthous H (2021). Scientific rationale for developing potent RBD-based vaccines targeting COVID-19. NPJ Vaccines.

[80] Burnett DL (2021). Immunizations with diverse sarbecovirus receptor-binding domains elicit SARS-CoV-2 neutralizing antibodies against a conserved site of vulnerability. Immunity.

[81] Deshpande A, Harris BD, Martinez-Sobrido L, Kobie JJ, Walter MR (2021). Epitope classification and RBD binding properties of neutralizing antibodies against SARS-CoV-2 variants of concern. Front. Immunol..

[82] Haynes WA (2021). High-resolution epitope mapping and characterization of SARS-CoV-2 antibodies in large cohorts of subjects with COVID-19. Commun. Biol..

[83] Smirnov D, Schmidt JJ, Capecchi JT, Wightman PD (2011). Vaccine adjuvant activity of 3M-052: An imidazoquinoline designed for local activity without systemic cytokine induction. Vaccine.

[84] Kastenmuller K (2011). Protective T cell immunity in mice following protein-TLR7/8 agonist-conjugate immunization requires aggregation, type I IFN, and multiple DC subsets. J. Clin. Investig..

[85] Thuluva S (2022). Evaluation of safety and immunogenicity of receptor-binding domain-based COVID-19 vaccine (Corbevax) to select the optimum formulation in open-label, multicentre, and randomised phase-1/2 and phase-2 clinical trials. EBioMedicine.

[86] An Y (2022). A tandem-repeat dimeric RBD protein-based covid-19 vaccine zf2001 protects mice and nonhuman primates. Emerg. Microbes Infect..

[87] Arunachalam PS (2021). Adjuvanting a subunit COVID-19 vaccine to induce protective immunity. Nature.

[88] Toback S (2022). Safety, immunogenicity, and efficacy of a COVID-19 vaccine (NVX-CoV2373) co-administered with seasonal influenza vaccines: an exploratory substudy of a randomised, observer-blinded, placebo-controlled, phase 3 trial. Lancet Respir. Med..

[89] Pino M (2021). A yeast expressed RBD-based SARS-CoV-2 vaccine formulated with 3M–052-alum adjuvant promotes protective efficacy in non-human primates. Sci. Immunol..

[90] Saunders KO (2021). Neutralizing antibody vaccine for pandemic and pre-emergent coronaviruses. Nature.

[91] Allen JC, Toapanta FR, Chen W, Tennant SM (2020). Understanding immunosenescence and its impact on vaccination of older adults. Vaccine.

[92] Sette A, Crotty S (2021). Adaptive immunity to SARS-CoV-2 and COVID-19. Cell.

[93] Tarke A (2021). Impact of SARS-CoV-2 variants on the total CD4(+) and CD8(+) T cell reactivity in infected or vaccinated individuals. Cell Rep. Med..

[94] Lee J (2021). Process development and scale-up optimization of the SARS-CoV-2 receptor binding domain-based vaccine candidate, RBD219-N1C1. Appl. Microbiol. Biotechnol..

[95] Stegmann T (2010). Lipopeptide-adjuvanted respiratory syncytial virus virosomes: A safe and immunogenic non-replicating vaccine formulation. Vaccine.

[96] Hyseni I (2020). Characterisation of SARS-CoV-2 lentiviral pseudotypes and correlation between pseudotype-based neutralisation assays and live virus-based micro neutralisation assays. Viruses.

[97] Manenti A (2020). Evaluation of SARS-CoV-2 neutralizing antibodies using a CPE-based colorimetric live virus micro-neutralization assay in human serum samples. J. Med. Virol..

[98] Wolfel R (2020). Virological assessment of hospitalized patients with COVID-2019. Nature.

[99] Salguero FJ (2021). Comparison of rhesus and cynomolgus macaques as an infection model for COVID-19. Nat. Commun..

[100] Cirelli KM (2019). Slow delivery immunization enhances HIV neutralizing antibody and germinal center responses via modulation of immunodominance. Cell.

