# Supplementary material for: A low dose of RBD and TLR7/8 agonist displayed on influenza virosome particles protects rhesus macaque against SARS-CoV-2 challenge
Source: Sci Rep. 2023 Mar 28;13:5074. doi: 10.1038/s41598-023-31818-y (PMC10044094; doi:10.1038/s41598-023-31818-y)
Supplement: Supplementary file 1 — Supplementary Information. [file 41598_2023_31818_MOESM1_ESM.pdf]

A Low Dose of RBD and TLR7/8 Agonist Displayed on Influenza Virosome Particles Protects Rhesus Macaque Against SARS-CoV-2 challenge.

Gerrit Koopman[1], Mario Amacker[2,3], Toon Stegmann[4], Ernst J. Verschoor[1], Babs E. Verstrepen[1], Farien Bhoelan[4], Denzel Bemelman[4], Kinga P. Böszörményi[1], Zahra Fagrouch[1], Gwendoline Kiemenyi-Kayere[1], Daniella Mortier[1], Dagmar E. Verel[1], Henk Niphuis[1], Roja Fidel Acar[1], Ivanela Kondova[5], Yolanda S. Kap[1], Willy M. J. M. Bogers[1], Petra Mooij[1], and Sylvain Fleury[2]*

[1]Department of Virology, Biomedical Primate Research Centre (BPRC), Rijswijk, Netherlands.

[2]Mymetics SA, 4 route de la Corniche, 1066 Epalinges, Switzerland.

[3]Department for BioMedical Research DBMR, Department of Pulmonary Medicine, Inselspital, Bern University Hospital, University of Bern, 3008 Bern, Switzerland

[4]Mymetics BV, JH Oortweg 21, 2333 CH Leiden, The Netherlands.

[5]Animal Science Department, Biomedical Primate Research Centre (BPRC), Rijswijk, Netherlands

*Corresponding author: Sylvain Fleury, Mymetics SA, 4 route de la Corniche, 1066 Epalinges, Switzerland. Email address: sylvain.fleury@mymetics.com.

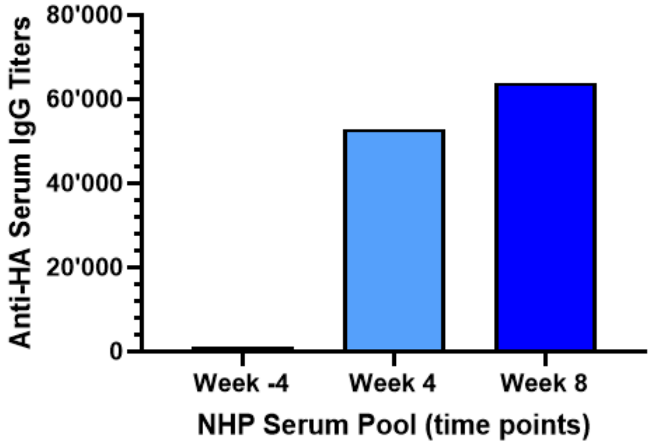

Supplementary Figure S1. ELISA serum IgG titers against influenza hemagglutinin (HA). NHP serum pools were collected at three different time points and evaluated in an ELISA coated with inactivated influenza virus A/Brisbane/59/2007 H1N1. Left bar: Day of intramuscular priming with inactivated influenza virus solution that corresponds to four weeks before vaccination (week -4). Middle bar: Four weeks after 1st vaccination performed with Virosome-RBD (week 4). Right bar: Four weeks after the second vaccination performed with Virosome-RBD (week 8). ELISA plates were coated with 1 mg HA/well for 16h at 4°C. After blocking and washing the wells, macaque serum IgGs bound to the HA were detected with a polyclonal goat anti-monkey IgG conjugated to horseradish peroxidase. After washing the plates, the TMB solution was added and the reaction was stopped by adding an acidic solution (H_2SO_4) that generates a yellow colour, and the optical density values were measure at 450 nm.

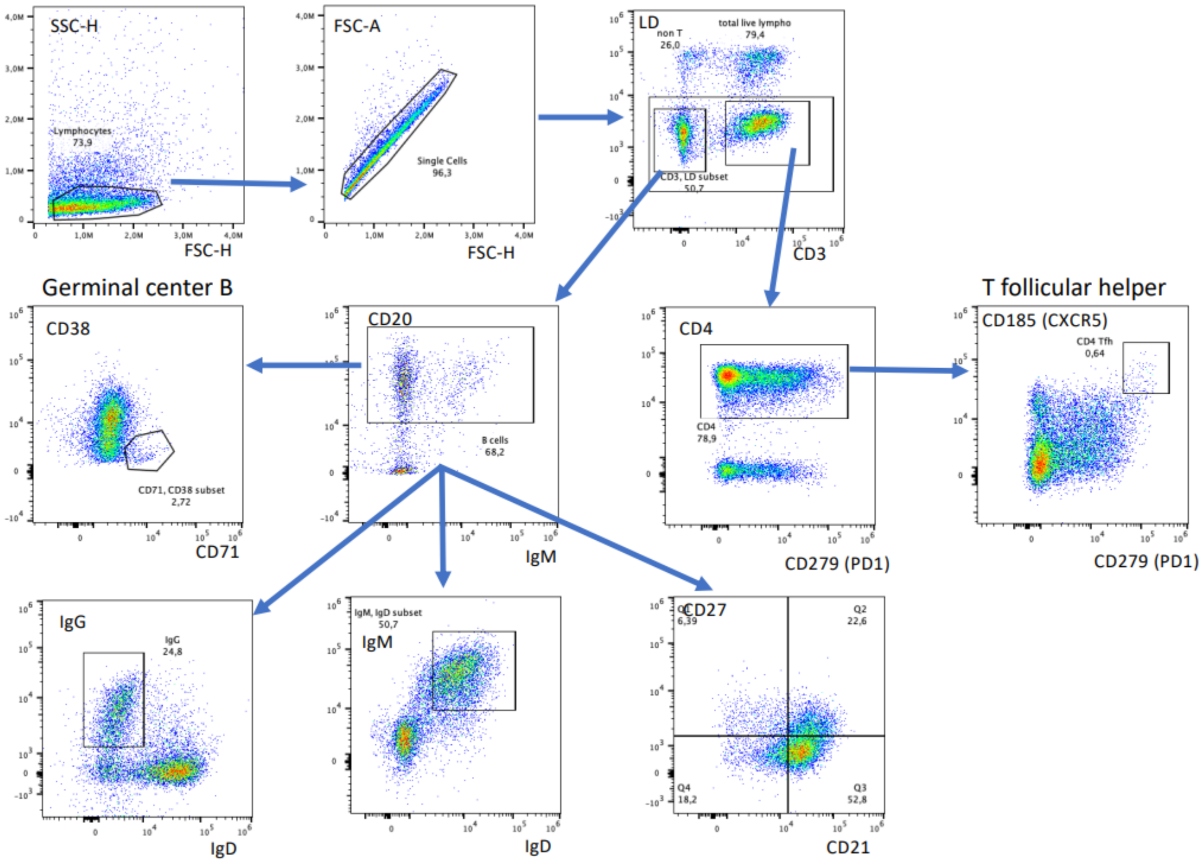

Supplementary Figure S2. Cytometry gating strategy on spleen cells collected at the day of euthanasia. During necropsy at day 7, 8 or 9 post challenge, the spleen was collected from the macaques. FACS analysis was performed on single cell suspensions that had been prepared from spleen obtained at autopsy. Thawed cells were first incubated with live/dead blue dead cell stain kit (Molecular Probes, cat. no. L23105). After 20 min. incubation, the cells were washed and then incubated with a mAb mixture containing: CD21^{BUV563} (BD bioscience, cat 741362), CD27^{BUV661} (BD, cat 741609), CD71^{B480} (BD, cat 746247), CD95^{BV711}(biolegend, cat 305644), CD38^{FITC} (Stem cell technologies, cat 10415), goat anti-human IgD^{Alexa555} (Southern Biotechnology Inc, cat 2030-32), CD297^{BV785} (PD1) (biolegend, cat 329930), CD185^{PE} (CXCR5) (Ebioscience, cat 12-9185-42), IgM$^{PerCP-Cy5.5}$ (BD, cat 561285), IgG^{APC} (BD, cat 550931), CD3^{Alexa700} (BD, cat 641414) diluted in Brilliant Stain Buffer Plus (BD, cat 566349). After 30 min incubation at 4°C in the dark, the cells were washed and fixed overnight at 4°C in 2% (w/v) paraformaldehyde solution in PBS. Flow cytometry was performed on an Aurora FACS machine using company software (Cytek, Fremont, CA, USA). For each tube the maximum number of events were recorded. Germinal center (GC) B cells and T follicular helper (Tfh) cells were analyzed by selecting cells within the lymphocyte gate, and then the singlets by using the forward scatter area plotted against the forward scatter height. Subsequently dead cells were excluded, and GC were identified as: CD3^{neg}/CD20^{pos}/CD38^{neg}/CD71^{pos} cells, while Tfh were identified as CD3^{pos}/CD4^{pos}CD279 (PD1)^{bright}/CD185 (CXCR5)^{pos}.

Table S1. Clinical chemistry parameters for evaluating the virosomes-RBD/3M-052 vaccine safety and tolerance.

	Parameter	Albumin	Alkaline Phosphatase	Alanine amino-transferase	Amylase	Bilirubin total	Urea	Calcium	Phosphate	Creatinin	Glucose	Sodium	Potassium	Total Protein	Globulin
	Abreviation	ALB	ALP	ALT	AMY	TBIL	BUN Urea	CA	PHOS	CRE	GLU	NA+	K+	TP	GLOB
	Unit	g/L	U/L	U/L	U/L	umoll/L	mmoll/L	mmoll/L	mmoll/L	umoll/L	mmoll/L	mmoll/L	mmoll/L	g/L	g/L
	Normal low	42	73	20	146	4	3.4	2.31	0.65	39	3.7	141	3.6	59	14
	Normal high	53	498	153	467	6.42	10.1	2.72	2.01	119	9.8	154	5.2	76	28
Animal week -4 Flu priming	V1	51	114	32	341	6	4.3	2.54	0.92	82	6	147	4.6	65	16
	V2	57	114	32	341	6	4.3	2.54	0.92	82	6	147	4.6	65	17
	V3	49	114	32	341	6	4.3	2.54	0.92	82	6	147	4.6	65	16
	V4	52	114	32	341	6	4.3	2.54	0.92	82	6	147	4.6	65	19
	V5	53	114	32	341	6	4.3	2.54	0.92	82	6	147	4.6	65	20
	V6	52	114	32	341	6	4.3	2.54	0.92	82	6	147	4.6	65	17
	C1	50	114	32	341	6	4.3	2.54	0.92	82	6	147	4.6	65	17
	C2	52	114	32	341	6	4.3	2.54	0.92	82	6	147	4.6	65	16
	C3	52	94	35	214	5	4.5	2.51	1.34	96	8.1	144	4	70	17
	C4	53	106	33	210	5	4.6	2.55	1.23	126	7.2	145	3.7	72	19
Animal week 4 after 1st vaccine	V1	50	103	107	256	5	3.8	2.48	1.42	96	5.3	143	4.2	66	16
	V2	55	117	30	254	5	4.8	2.56	1.12	87	6.1	142	4.3	71	16
	V3	49	108	90	253	5	4.2	2.49	1.06	75	8.2	141	4.1	66	17
	V4	50	164	43	497	5	5.4	2.52	0.91	132	7	145	3.6	67	17
	V5	54	158	27	339	6	4.4	2.5	1.76	104	5.6	147	4	74	20
	V6	52	237	58	460	6	5.8	2.52	1.76	111	6.1	150	3.6	69	17
	C1	52	94	35	214	5	4.5	2.51	1.34	96	8.1	144	4	70	17
	C2	53	106	33	210	5	4.6	2.55	1.23	126	7.2	145	3.7	72	19
	C3	49	111	25	242	9	3.9	2.47	1.25	92	6.1	147	4	66	17
	C4	51	389	35	165	5	5.4	2.52	1.11	115	8.2	144	3.7	66	15
Animal week 6 / week 2 after 2nd vaccine	V1	48	88	69	244	5	4.8	2.51	1.7	86	7	149	4.1	64	16
	V2	53	103	22	252	5	4.7	2.66	1.39	65	6.5	154	4.4	70	17
	V3	46	97	71	242	5	5	2.58	1.36	79	7.3	152	3.9	66	20
	V4	49	128	35	299	3	8	2.57	1.28	126	7.2	153	4	67	18
	V5	52	144	24	318	5	4.9	2.6	1.65	99	7.4	155	3.8	71	19
	V6	53	182	64	329	4	6	2.59	1.71	98	7.4	149	3.6	75	23
	C1	49	104	29	270	4	4.4	2.54	1.5	104	9.9	150	3.8	67	18
	C2	48	309	30	149	5	6.7	2.57	1.44	115	9.4	146	3.8	64	16
	C3	49	104	29	270	4	4.4	2.54	1.5	104	9.9	150	3.8	67	18
	C4	48	309	30	149	5	6.7	2.57	1.44	115	9.4	146	3.8	64	16

Table S2. Hematology parameters for evaluating the virosomes-RBD/3M-052 vaccine safety and tolerance.

	Parameter	White blood cell count	Lymphocyte count	Monocyte count	Neutrohil count	% Lymphocytes	% Monocytes	% Neutrophils	Red blood cell count	Hemoglobin	Hematocrit	Mean Corpuscular Volume	Mean Corpuscular Hemoglobin	Mean Corpuscular Hemoglobin Concentration	Red Blood cell distribution Width-cv	Red Blood cell distribution Width-sd	Platelet count	Mean Platelet Volume	Plateletcrit	Platelet Distribution Width-cv	Platelet Distribution Width-sd
	Abreviation	WBC	LYM	MON	NEU	% Lymp.	% Mon.	% Neu.	RBC	HGB	HCT	MCV	MCH	MCHC	RDWc	RDWs	PLT	MPV	PCT	PDWc	PDWs
	Unit	10E9/L	10E9/L	10E9/L	10E9/L	%	%	%	10E12/L	mmoll/L	%	fl	amol	mmoll/L	%	fl	10E9/L	fl	%	%	fl
	Normal low	4.0	1.0	0.0	0.2	11.3	0.5	3.8	5.3	7.2	39.0	67	1240.8	18.0	14.6	39.8	235.3	8.0	0.3	35.0	11.8
	Normal high	14.5	6.0	4.3	10.3	63.3	46.2	79.1	7.1	9.7	49.7	77	1541.1	20.6	16.7	46.1	523.9	12.4	0.5	41.8	21.8
Animal week -4 Flu priming	V1	6.4	3.2	0.2	1.9	50.3	2.3	30.3	6.3	8.8	45.1	72.0	1390.0	19.4	16.0	43.8	332.0	10.1	0.3	38.7	15.9
	V2	9.2	3.2	0.1	4.2	34.8	0.6	45.5	6.5	8.7	47.1	72.0	1330.0	18.5	15.8	43.0	251.0	11.5	0.3	40.1	20.7
	V3	8.6	1.8	0.1	5.8	21.4	0.9	67.8	6.5	8.8	47.0	73.0	1370.0	18.8	15.2	42.2	423.0	8.7	0.4	36.7	13.5
	V4	13.6	3.6	0.1	8.6	26.7	0.5	62.7	6.1	8.5	43.6	71.0	1380.0	19.4	14.8	39.8	397.0	11.2	0.4	39.7	18.6
	V5	7.4	3.2	0.1	3.1	43.1	0.8	42.2	5.8	8.3	42.7	74.0	1430.0	19.4	15.4	43.0	422.0	10.3	0.4	38.2	16.4
	V6	6.0	2.9	0.1	2.4	48.2	1.0	40.5	6.2	8.7	43.7	71.0	1410.0	19.9	15.0	40.6	384.0	10.5	0.4	38.2	16.4
	C1	7.6	2.4	0.1	4.2	32.1	1.0	55.7	6.4	8.9	47.3	74.0	1380.0	18.7	16.5	46.1	297.0	9.6	0.3	37.8	14.8
	C2	10.6	4.5	0.1	4.8	42.8	0.9	45.6	6.5	8.9	46.2	71.0	1370.0	19.1	15.6	42.2	363.0	9.8	0.4	36.9	14.8
	C3	5.4	2.2	0.1	2.6	41.2	1.5	47.9	6.4	9.1	45.7	71.0	1410.0	19.9	15.3	41.4	239.0	10.3	0.3	39.1	17.8
	C4	6.2	2.6	0.1	3.1	41.8	1.0	49.9	6.7	9.2	47.6	71.0	1370.0	19.4	15.9	43.0	261.0	11.2	0.3	40.0	19.1
Animal week 4 after 1st vaccine	V1	8.2	4.0	0.0	3.2	48.8	0.5	38.7	10.6	16.6	75.3	71.0	1.6	22.1	17.3	46.1	531.0	10.5	0.6	38.9	16.1
	V2	9.9	5.0	0.1	3.8	50.5	0.7	38.7	11.0	17.4	76.7	70.0	1.6	22.7	16.7	43.8	291.0	11.5	0.3	41.7	22.3
	V3	4.3	0.9	0.1	2.9	21.0	1.4	67.0	7.3	10.6	54.0	74.0	1.5	19.6	15.4	43.0	269.0	8.9	0.2	37.2	14.0
	V4	12.5	2.3	0.1	9.5	18.2	0.6	75.6	11.0	17.4	80.0	73.0	1.6	21.8	16.5	45.3	556.0	11.9	0.7	42.8	23.1
	V5	11.1	4.8	0.1	5.8	43.1	0.7	52.6	10.8	17.4	79.6	74.0	1.6	21.9	16.5	45.3	825.0	11.0	0.9	40.5	18.6
	V6	7.4	1.3	0.1	5.4	17.6	0.9	72.8	11.3	18.1	79.9	71.0	1.6	22.6	17.1	45.3	478.0	10.5	0.5	39.1	16.4
	C1	5.4	2.2	0.1	2.6	41.2	1.5	47.9	6.4	9.1	45.7	71.0	1410.0	19.9	15.3	41.4	239.0	10.3	0.3	39.1	17.8
	C2	6.2	2.6	0.1	3.1	41.8	1.0	49.9	6.7	9.2	47.6	71.0	1370.0	19.4	15.9	43.0	261.0	11.2	0.3	40.0	19.1
	C3	12.1	3.9	0.1	6.9	32.1	1.1	57.4	10.5	15.8	73.7	70.0	1.5	21.4	16.7	44.5	544.0	10.7	0.6	38.9	16.1
	C4	18.3	6.9	0.1	8.5	37.5	0.7	46.2	10.3	15.9	75.4	73.0	1.5	21.1	16.7	46.1	664.0	10.1	0.7	38.9	16.1
Animal week 6 / week 2 after 2nd vaccine	V1	4.9	1.9	0.0	2.3	39.0	0.6	46.3	6.4	8.9	46.7	74.0	1400.0	19.1	16.1	44.5	270.0	10.1	0.3	38.5	15.6
	V2	4.9	1.6	0.0	2.9	33.2	6.0	59.0	5.0	8.9	36.3	72.0	1770.0	24.5	15.0	40.6	257.0	10.4	0.3	39.0	17.5
	V3	9.1	2.9	0.1	4.9	31.5	0.7	54.3	6.1	8.5	45.2	74.0	1390.0	18.9	15.8	43.8	410.0	9.4	0.4	37.4	14.3
	V4	7.8	1.5	0.1	5.6	19.8	0.8	72.5	5.9	8.0	42.9	73.0	1350.0	18.6	15.2	41.4	256.0	11.3	0.3	39.7	18.6
	V5	5.5	1.9	0.0	3.0	35.0	0.5	55.1	5.7	7.6	41.6	73.0	1350.0	18.4	15.4	43.0	286.0	10.3	0.3	37.8	15.9
	V6	4.7	1.6	0.1	2.8	34.7	1.5	58.7	5.1	8.5	36.8	73.0	1670.0	23.0	15.2	41.4	252.0	10.1	0.3	38.2	16.4
	C1	9.3	3.1	0.2	4.8	33.7	2.3	52.2	6.2	8.8	45.0	72.0	1200.0	16.7	15.2	42.2	360.0	10.2	0.4	37.8	15.9
	C2	9.3	2.9	0.1	5.2	31.5	0.9	55.8	6.2	8.8	45.6	74.0	1430.0	19.4	16.0	44.5	389.0	9.0	0.4	36.7	13.5
	C3	9.3	3.1	0.2	4.8	33.7	2.3	52.2	6.2	8.8	45.0	72.0	1200.0	16.7	15.2	42.2	360.0	10.2	0.4	37.8	15.9
	C4	9.3	2.9	0.1	5.2	31.5	0.9	55.8	6.2	8.8	45.6	74.0	1430.0	19.4	16.0	44.5	389.0	9.0	0.4	36.7	13.5
